# SLEMM: million-scale genomic predictions with window-based SNP weighting

**DOI:** 10.1093/bioinformatics/btad127

**Published:** 2023-03-10

**Authors:** Jian Cheng, Christian Maltecca, Paul M VanRaden, Jeffrey R O'Connell, Li Ma, Jicai Jiang

**Affiliations:** Department of Animal Science, North Carolina State University, Raleigh, NC 27695, United States; Department of Animal Science, North Carolina State University, Raleigh, NC 27695, United States; Animal Genomics and Improvement Laboratory, USDA-ARS, Beltsville, MD 20705, United States; Department of Medicine, University of Maryland School of Medicine, Baltimore, MD 21201, United States; Department of Animal and Avian Sciences, University of Maryland, College Park, MD 20742, United States; Department of Animal Science, North Carolina State University, Raleigh, NC 27695, United States

## Abstract

**Motivation:**

The amount of genomic data is increasing exponentially. Using many genotyped and phenotyped individuals for genomic prediction is appealing yet challenging.

**Results:**

We present SLEMM (short for Stochastic-Lanczos-Expedited Mixed Models), a new software tool, to address the computational challenge. SLEMM builds on an efficient implementation of the stochastic Lanczos algorithm for REML in a framework of mixed models. We further implement SNP weighting in SLEMM to improve its predictions. Extensive analyses on seven public datasets, covering 19 polygenic traits in three plant and three livestock species, showed that SLEMM with SNP weighting had overall the best predictive ability among a variety of genomic prediction methods including GCTA’s empirical BLUP, BayesR, KAML, and LDAK’s BOLT and BayesR models. We also compared the methods using nine dairy traits of ∼300k genotyped cows. All had overall similar prediction accuracies, except that KAML failed to process the data. Additional simulation analyses on up to 3 million individuals and 1 million SNPs showed that SLEMM was advantageous over counterparts as for computational performance. Overall, SLEMM can do million-scale genomic predictions with an accuracy comparable to BayesR.

**Availability and implementation:**

The software is available at https://github.com/jiang18/slemm.

## 1 Introduction

In modern animal and plant breeding, individuals are selected for breeding based on predictions of genetic merit. Pedigree-based predictions of genetic merits have been used for selection in animals and plants for many decades and achieved steady genetic progress. With the advancement of sequencing technologies, millions of genetic markers (e.g. SNPs) have been discovered in the genome of many species. With the availability of cost-effective SNP chips, genomic predictions that use many genotyped SNPs across the genome (Meuwissen et al. 2001) have been widely applied to animals and plants to improve genetic progress (Bernardo and Yu 2007; VanRaden et al. 2009) and to humans to predict polygenic risks of diseases (Chatterjee et al. 2016). Genomic selection is particularly attractive for complex traits with a low heritability that are often underlain by many loci with small effects (Goddard and Hayes 2009). One common method for genomic predictions is the genomic best linear unbiased prediction (GBLUP), which assumes that SNP effects are independent and identically distributed normal variables (Meuwissen et al. 2001; VanRaden 2008). This assumption, however, can adversely affect the predictions where major QTLs or genes underlie a trait. Various Bayesian methods that assume different prior distributions of SNP effects, such as BayesA, BayesB, and BayesR, have been developed and proven to outperform GBLUP for predicting phenotypes underlain by major QTLs (Meuwissen et al. 2001; Moser et al. 2015). BayesR is one of the widely used Bayesian methods, which assumes that SNP effects follow a mixture of four zero-mean normal distributions with relative variance weights of 0, 0.01, 0.1, and 1 (Moser et al. 2015). Bayesian methods can have flexible priors on SNP effects; however, the Markov chain Monte Carlo (MCMC) algorithms often used by these Bayesian methods are time consuming.

It is appealing to tackle the computational challenge of Bayesian methods but maintain their advantage over GBLUP. One way is to implement the Bayesian methods with variational Bayes, e.g. LDAK (Zhang et al. 2021). Alternatively, many studies have attempted to weight SNPs to enable weighted GBLUP (Tiezzi and Maltecca 2015; Zhang et al. 2015, 2016; Teissier et al. 2018; Mehrban et al. 2021; Zhang et al. 2015, 2016). In particular, Yin et al. (2020) proposed a machine-learning-based method named KAML, which can optimize the SNP weights and fit large-effect SNPs as fixed effects in the GBLUP model. KAML outperformed GBLUP and had comparable accuracies to BayesR (Yin et al. 2020); however, this method has not been tested using a large dataset. The amount of genomic data is increasing exponentially in animal and plant breeding and in human disease risk prediction. It is appealing yet challenging to use millions of genotyped and phenotyped individuals for genomic predictions.

This article presents a new software tool to address the challenge, which we refer to as SLEMM (short for Stochastic-Lanczos-Expedited Mixed Models). SLEMM can efficiently perform Stochastic Lanczos Restricted Maximum Likelihood (REML) analysis (Border and Becker, 2019) for millions of genotyped individuals and weight SNPs based on proximity and minor allele frequency (MAF). Because of linkage disequilibrium (LD) between SNPs, effect sizes of SNPs that are close to each other tend to be similar in model fitting (Yang and Tempelman 2012; Zeng et al. 2018), which stimulates proximity-based SNP weighting. SNP heritability estimation can be affected by the assumption of the relationship between MAF and SNP effect size (Speed et al. 2017). Such a relationship may be modeled to improve genomic predictions. We evaluated SLEMM with seven public datasets covering multiple animal and plant species, a large dairy cow dataset (300k animals) from the U.S. Council of Dairy Cattle Breeding (CDCB), and large simulation data. We compared SLEMM with GBLUP implemented in GCTA (Yang et al. 2015), BayesR (one of the fastest MCMC methods), LDAK (featuring variational Bayes implementation of mixture models), and KAML (highlighting machine learning), and demonstrated that SLEMM can do million-scale genomic predictions with an accuracy comparable to BayesR.

## 2 Materials and methods

### 2.1 Statistical models

SLEMM fits the following linear mixed model:


(1) $$\begin{array}{c} y=X\beta +Z\alpha +e\\ \alpha ∼N(0,W\sigma_{\alpha }^{2})\\ e∼N(0,R\sigma_{e}^{2}) \end{array},$$

where **y** is a vector of phenotypes for a quantitative trait, **β** is a vector of fixed effects including mean, **X** is the design matrix for **β**, **α** is a vector of SNP effects with a diagonal covariance matrix $W\sigma_{\alpha }^{2}$, **Z** is a matrix of standardized genotypes, and **e** is a vector of residuals with diagonal covariance matrix $R\sigma_{e}^{2}$. **R** is often equal to an identity matrix; however, when using deregressed estimated breeding values as **y** in a farm animal population, we need to precompute **R** to model their reliability (VanRaden 2008). Diagonal elements of **W** are weights with a mean of one for SNP effect variance; that is, *W_jj_* represents the relative contribution of SNP *j* to genetic variance. Optimal SNP weighting can improve genomic prediction.

### 2.2 SNP weighting

We provide two schemes to optimize SNP weighting for genomic predictions. The first scheme is based on the MAF dependence of SNP effect sizes, which has been well studied for complex traits in humans (Yang et al. 2015; Speed et al. 2017; Schoech et al. 2019). It can be represented by the probability density function of the beta distribution (Wu et al. 2011): where *C* is a scaling constant for achieving a mean weight of one, *p_j_* is the MAF of SNP *j*, and *a* and *b* are two shape parameters of the beta distribution. As in the sequence kernel association test (Wu et al. 2011), setting 0 < *a *≤* *1 and *b *≥* *1 can be used to increase the weight of rarer variants and decrease the weight of more common variants if rarer variants are expected to have larger effects. Setting *a *=* b *=* *1 denotes that all SNPs equally contribute to heritability regardless of their MAFs. It is common to assume that an SNP’s contribution to heritability is proportional to 2*p*(1−*p*) in animal breeding, which is equivalent to *a *=* b *=* *2 in our method. We can use grid search to determine the optimal values of *a* and *b* based on REML log-likelihood. In practice, we can set *a *=* b* and search among a small set of values (e.g. {0.5, 1, 1.5, 2, 2.5} or {1, 1.25, 1.5, 1.75, 2}) to reduce search space.


(2) $$W_{jj}=C\cdot p_{j}^{a-1}{\left(1-p_{j}\right)}^{b-1},$$

Our second SNP weighting scheme is based on estimates of SNP effects with **W** equal to the identity. A QTL’s effect can be captured by its nearby SNPs because of LD, and SNPs in proximity to each other tend to show similar effects in model fitting. This leads us to develop the following window-based weighting scheme: where *C* is defined the same as in equation (2), *S* is the number of SNPs on each side of SNP *j*, and ${\hat{\alpha }}_{k}$ is the estimate of *k*th SNP’s effect in an existing BLUP with **W** equal to the identity. This specification of *j*th SNP’s weight borrows information from a window of 2*S *+* *1 SNPs. It is similar to the window-based weighting used in weighted ssGBLUP (Zhang et al. 2016). SLEMM fits model (1) with training data twice, first with **W** equal to identity and second with **W** computed by equation (3).


(3) $$W_{jj}=C\cdot \frac{1}{2S+1}\sum_{k=j-S}^{j+S}{\hat{\alpha }}_{k}^{2},$$

Assuming that the two SNP weighting schemes produce independent weights, we can combine them using in which all parameters are the same as defined in equations (2) and (3). In this case, SLEMM fits model (1) with training data twice, first with **W** equal to identity and second with **W** computed by equation (4).


(4) $$W_{jj}=C\cdot p_{j}^{a-1}{\left(1-p_{j}\right)}^{b-1}\cdot \frac{1}{2S+1}\sum_{k=j-S}^{j+S}{\hat{\alpha }}_{k}^{2}$$

### 2.3 Stochastic Lanczos algorithms and software

SLEMM builds on efficient REML for model (1). Following Border and Becker (2019), we applied the stochastic Lanczos REML (SL-REML) algorithms in the linear mixed model. Compared to the previous SL-REML method (Border and Becker 2019), SLEMM allows **W** and **R** to be non-identity, which is important for genomic prediction, particularly in animal breeding. Given the REML estimates of variance components, it is straightforward to compute BLUPs of SNP effects. Details on the algorithms are provided in the Supplementary Methods.

SLEMM is written in C++ and powered by the Eigen library. It uses bool vectors to store genotypes in memory, occupying two bits per SNP per individual. SLEMM has a time complexity of *O*(*mn*^1.5^) and uses <mn/2 bytes for *n* individuals and *m* SNPs.

### 2.4 Benchmarking

We compared SLEMM to GCTA’s empirical GBLUP (Yang et al. 2015), KAML (Yin et al. 2020), BayesR (specifically, BayesRv2 at https://github.com/syntheke/bayesR) (Moser et al. 2015), and LDAK’s BOLT and BayesR methods (Zhang et al. 2021). GCTA’s empirical GBLUP assumes that all SNPs contribute equally to SNP heritability. KAML fits large SNPs effects as fixed in a linear mixed model and optimizes SNP weights in the genomic relationship matrix (GRM) using a machine learning method. BayesR uses a Bayesian mixture model along with a computationally optimized Gibbs sampling algorithm, which assumes that SNP effects follow a mixture of four zero-mean normal distributions with relative variance weights of 0, 0.01, 0.1, and 1. LDAK-BayesR is based on largely the same model as BayesR but uses a variational Bayes method instead of Gibbs sampling to fit the model. LDAK-BOLT adopts the prediction method of BOLT-LMM (Loh et al. 2015), which is based on a mixture of two zero-mean normal distributions, one with a relatively small variance and the other with a relatively large variance. Both the LDAK prediction methods use grid search and cross-validation to optimize the hyper-parameters in the mixture priors. They have two versions, one for using individual data and the other for using summary statistics. In addition, LDAK can use pre-specified weights for individual SNPs in the predictions. In our analysis, we used the versions for individual data and MAF-based SNP weighting.

We applied the following key settings to all our analyses: optimized MAF-based SNP weighting for SLEMM-MAFopt; a fixed window size of 20 and optimized MAF-based SNP weighting for SLEMM-WW-MAFopt; defaults for KAML; blocksize = 4, msize = 500, number of MCMC iterations = 50 000, and burn-in = 20 000 iterations for BayesR; and defaults for LDAK with MAF-based SNP weighting the same as SLEMM-MAFopt.

We used repeated random sub-sampling validation to evaluate the predictive ability of the aforementioned methods with seven public datasets. Each dataset was randomly split into a training population (about 80% of individuals) and a validation population (about 20% of individuals) for 20 replicates. Five-fold cross-validation was used for the large dairy cow data to reduce computational time. Predictive ability was calculated as the Pearson correlation in the validation population between genomic estimated breeding values (GEBVs) and pre-adjusted phenotypes or (de-regressed) EBVs.

Computational speed and memory usage were evaluated using simulated data for SLEMM, KAML, BayesR, and LDAK. All evaluations were conducted on a Linux server with 2x Intel(R) Xeon(R) Gold 6258R CPU and 512 GB of memory. Ten threads were used for SLEMM, KAML, and LDAK, whereas four threads (=block size) were used for BayesR. Ten threads were tried for BayesR, but it slowed down the computation and sometimes led to failure.

### 2.5 Real data

We analyzed 19 livestock and plant traits in seven public datasets and nine dairy traits in a large cow dataset from the U.S. CDCB. A summary of the datasets is shown in Table 1, and a detailed description on each dataset is available in the Supplementary Methods.

**Table 1.** Summary of datasets.

Species	Sample size	Trait ([pseudo-] heritability)	Genotype (# SNPs)	Genotyping platform	Reference
Dairy bull	5024	MY (0.94), MFP (0.95), SCS (0.88)	42 551	Illumina Bovine SNP50 Beadchip	Zhang et al. (2015)
Duroc pig	2785	BF (0.25), LMD (0.35), TTN (0.30)	49 564	Low-coverage whole-genome sequencing	Yang et al. (2021)
Four pig breeds	4260	ADG (0.49), AGE (0.51)	47 157	Illumina PorcineSNP50 Beadchip	Tang et al. (2019)
Chicken	1063	EW1 (0.31), EW36 (0.36), EW56 (0.23)	44 430	Affymetrix 600K chicken SNP chip	Liu et al. (2018)
Maize	1868	Sweet (0.60), GDD (0.70)	45 426	Genotyping by sequencing	Romay et al. (2013)
Wheat	599	GY1 (0.29), GY2 (0.26), GY3 (0.28), GY4 (0.27)	1,279	Diversity Array Technology (DArT)	http://cran.r-project.org/web/packages/BLR/index.html
Pine	861	DBH (0.31), HT (0.31)	4853	Illumina Infinium assay	Resende et al. (2012)
Dairy cow	294 079	Milk (0.34), Fat (0.31), Pro (0.27), FPC (0.42), PPC (0.44),	60 671	Imputed from 18 SNP chips with 2710 to 60 671 original SNPs	Jiang et al. (2019)
		SCS (0.10), DPR (0.04), CCR (0.05), HCR (0.01)			

MY, milk yield; MFP, milk fat percentage; SCS, somatic cell score; BF, backfat; LMD, loin muscle depth; TTN, total teat number; ADG, average daily gain; AGE, off-test age; EW1, first egg weight; EW36, egg weight at 36 weeks old; EW56, egg weight at 56 weeks old; Sweet, kernel is sweet or starchy; GDD, growing degree days; GY1, grain yield in environment 1; GY2, grain yield in environment 2; GY3, grain yield in environment 3; GY4, grain yield in environment 4; DBH, stem diameter; HT, total stem height; Milk, milk yield; Fat, fat yield; Pro, protein yield; FPC, milk fat percentage; PPC, milk protein percentage; CCR, cow conception rate; HCR, heifer conception rate; DPR, daughter pregnancy rate.

### 2.6 Simulation

We simulated two datasets, one with 3M unrelated animals and 50k SNPs and the other with 1M SNPs and 100k unrelated animals. The former dataset was used to evaluate the computational performance with respect to sample size (100k, 200k, 300k, 400k, 500k, 1M, and 3M animals), while the latter dataset was used to evaluate the computational performance with respect to the number of SNPs (200k, 400k, 600k, 800k, and 1M SNPs). Genotypes were simulated using the software genosim (VanRaden et al. 2015) with default input parameters; in particular, we set LDparam = 0.965 to mimic a high level of LD. We randomly selected 5000 SNPs as QTLs and sampled QTL effects from the standard normal distribution. Phenotypes with a heritability of 0.3 were simulated by adding the additive genetic effects and residual effects.

## 3 Results

Considering the involvedness of the genetic architecture of complex traits, we used only real data to evaluate prediction accuracy, including 19 traits from publicly available plant and livestock data and 9 traits of 300k dairy cows from the U.S. CDCB. We used simulation to benchmark computing time and memory usage. We considered three types of empirical GBLUP analyses in SLEMM: (i) non-weighted GBLUP (SLEMM-NW) that assumes all SNPs contribute equally to SNP heritability without SNP weighting, (ii) SLEMM-MAFopt that optimizes SNP weighting based on MAF, and (iii) SLEMM-WW-MAFopt that weights SNPs based on both MAF and proximity. We compared SLEMM to non-weighted empirical GBLUP implemented in GCTA (GCTA-NW), KAML, BayesR, and two LDAK models (LDAK-BayesR and LDAK-BOLT).

### 3.1 Public livestock and plant data

Table 2 shows the prediction accuracies for 11 traits in four livestock (dairy bull, pig, and chicken) datasets and eight traits in three plant (maize, wheat, and pine) datasets (see Supplementary Figs S1–S7 for each individual dataset). BayesR performed abnormally for the dataset of four pig breeds and the maize dataset, and KAML failed in the maize data. So, the two datasets were excluded from the software comparison. SLEMM-NW had exactly the same prediction accuracy as GCTA-NW, which is expected because they are the same non-weighted empirical GBLUP. SLEMM-WW-MAFopt, KAML, BayesR, LDAK-BayesR, and LDAK-BOLT (with an overall accuracy of 0.489, 0.486, 0.492, 0.489, and 0.488, respectively) outperformed SLEMM-NW (0.475) for livestock traits. SLEMM-WW-MAFopt (with an overall accuracy of 0.453) performed similarly to SLEMM-NW (0.452) for plant traits, and they both outperformed KAML (0.434), BayesR (0.441), LDAK-BayesR (0.422), and LDAK-BOLT (0.431). Additionally, SLEMM-MAFopt which optimizes SNP weighting based on MAF had an overall accuracy similar to SLEMM-NW for both livestock (0.480 versus 0.475) and plant (0.455 versus 0.452) traits. Collectively, SLEMM-WW-MAFopt had the best predictive ability.

**Table 2.** Average predictive ability across 20 replicates in repeated random sub-sampling validation for each trait in seven public datasets.

Trait	GCTA-NW (SD^a^)	SLEMM-NW	SLEMM-MAFopt	SLEMM-WW–MAFopt	KAML	BayesR	LDAK-BayesR	LDAK-BOLT
Dairy bull								
MY	0.766 (0.009)	0.766	0.770	0.784	0.785	0.786	0.780	0.783
MFP	0.803 (0.011)	0.803	0.816	0.865	0.862	0.870	0.856	0.860
SCS	0.737 (0.011)	0.737	0.739	0.742	0.741	0.740	0.739	0.738
Average	0.769	0.769	0.775	0.797	0.796	0.799	0.792	0.794
Duroc pig								
BF	0.321 (0.053)	0.321	0.321	0.327	0.321	0.329	0.321	0.318
LMD	0.345 (0.038)	0.345	0.345	0.343	0.351	0.354	0.350	0.352
TTN	0.388 (0.033)	0.388	0.392	0.405	0.413	0.415	0.408	0.403
Average	0.351	0.351	0.353	0.358	0.362	0.366	0.360	0.358
Four pig breeds								
ADG	0.572 (0.017)	0.572	0.572	0.564	0.567	–	0.568	0.571
AGE	0.563 (0.016)	0.563	0.563	0.555	0.557	–	0.559	0.561
Average	0.568	0.568	0.568	0.560	0.562	–	0.564	0.566
Chicken								
EW1	0.300 (0.053)	0.300	0.299	0.307	0.280	0.301	0.298	0.300
EW36	0.362 (0.066)	0.362	0.378	0.374	0.380	0.373	0.384	0.383
EW56	0.255 (0.059)	0.255	0.263	0.253	0.239	0.257	0.261	0.259
Average	0.306	0.306	0.313	0.311	0.300	0.310	0.314	0.314
Livestock average	0.475	0.475	0.480	0.489	0.486	0.492	0.489	0.488
Maize								
Sweet	0.895 (0.039)	0.895	0.895	0.900	–	–	0.898	0.900
GDD	0.904 (0.011)	0.904	0.900	0.899	–	–	0.888	0.896
Average	0.900	0.900	0.898	0.900	–	–	0.893	0.898
Wheat								
GY1	0.503 (0.063)	0.503	0.509	0.502	0.477	0.491	0.465	0.488
GY2	0.506 (0.052)	0.506	0.512	0.511	0.502	0.496	0.485	0.491
GY3	0.411 (0.071)	0.411	0.406	0.417	0.390	0.406	0.373	0.384
GY4	0.431 (0.051)	0.431	0.447	0.443	0.431	0.424	0.412	0.417
Average	0.463	0.463	0.469	0.468	0.450	0.454	0.434	0.445
Pine								
DBH	0.464 (0.052)	0.464	0.460	0.459	0.442	0.453	0.443	0.447
HT	0.394 (0.050)	0.394	0.394	0.384	0.362	0.377	0.351	0.359
Average	0.429	0.429	0.427	0.422	0.402	0.415	0.397	0.403
Plant average	0.452	0.452	0.455	0.453	0.434	0.441	0.422	0.431

a SD is the standard deviation of predictive ability across 20 replicates in repeated random sub-sampling validation for each trait. It was only provided for GCTA-NW because SD was similar across different methods.

### 3.2 CDCB dairy cow data

Table 3 shows the predictive ability of the methods in comparison for production, reproduction, and health traits in the large dairy cow data. LDAK-BayesR was not included in this comparison, because its predictive ability is close to that of LDAK-BOLT as shown in an earlier study (Zhang et al. 2021) and in our analysis of the public livestock and plant data. KAML failed to process such a large dataset. We particularly added ordinary least squares (OLS) for comparison, as it was feasible for the cow data. All the methods except OLS had overall similar predictive abilities. SLEMM-WW-MAFopt and LDAK slightly outperformed SLEMM-NW for milk fat and protein percentage traits. OLS had a much lower predictive ability than other methods for all the dairy traits.

**Table 3.** Average predictive ability of five-fold cross-validation for each trait in the large dairy cow data.^a^

Method	Production traits						Health	Reproduction traits			
	FPC	PPC	Milk	Fat	Pro	Average	SCS	CCR	HCR	DPR	Average
SLEMM-NW	0.636	0.659	0.479	0.449	0.425	0.530	0.243	0.136	0.073	0.160	0.123
SLEMM-MAFopt	0.636	0.659	0.479	0.449	0.426	0.530	0.243	0.135	0.073	0.160	0.123
SLEMM-WW-MAFopt	0.643	0.665	0.481	0.451	0.427	0.533	0.244	0.134	0.071	0.159	0.121
BayesR	0.641	0.654	0.481	0.450	0.426	0.530	0.243	0.135	0.073	0.160	0.123
LDAK-BOLT	0.642	0.663	0.480	0.450	0.426	0.532	0.243	0.135	0.073	0.160	0.123
OLS	0.544	0.571	0.357	0.319	0.295	0.417	0.118	0.029	0.012	0.031	0.024

a The sample size for training (80% of whole data) is ∼234 774 for production traits and SCS and ∼102 306 for reproduction traits.

### 3.3 SLEMM tuning

We evaluated the impact of MAF-based SNP weighting, window size, and the number of window-based SNP weighting rounds on genomic prediction accuracy in SLEMM. To find the optimal MAF weighting parameter in equation (2), we explored the relationship between REML log-likelihood and the MAF weighting parameter. It has been reported that the optimal MAF weighting parameter may be affected by large-effect QTLs (Speed et al. 2017). This was also observed in our analysis on the dairy bull data (Supplementary Fig. S8). Fitting significant SNP effects as fixed changed the optimal MAF weighting parameter value and made the trend across traits more consistent: REML log-likelihood peaked at around 1.5 for all three bull traits. As shown in Supplementary Fig. S9 on the dairy bull data, randomly selecting 80% of individuals as training data for 20 replicates resulted in largely the same relationship between the REML log-likelihood and the MAF weighting parameter. This suggests that the MAF weighting parameter is a robust parameter for each trait in a population. In addition, the large-scale dairy cow data analysis also showed that REML log-likelihood peaked at around 1.5 for all nine cow traits regardless of fitting significant SNP effects as fixed (Supplementary Fig. S10A) or not (Supplementary Fig. S10B).

Besides a window size of 20, we further tried a much larger window size (500 for livestock traits and 1000 for plant traits) in SLEMM-WW-MAFopt for all seven public datasets. The large window size resulted in similar predictive abilities to the window size of 20 for most traits (Supplementary Figs S11–S17). In particular, using the larger window size decreased the predictive ability for milk fat percentage in dairy bulls (Supplementary Fig. S11) but increased that for average daily weight gain and off-test age in the four-breed pig population (Supplementary Fig. S13).

The iterative weighting in SLEMM is set to one round by default. We further tried two rounds of window-based SNP weighting for the dairy bull traits. Increasing the number of weighting rounds marginally improved the predictive ability for MFP (0.864 versus 0.866) but decreased that for MY and SCS (Supplementary Fig. S18).

### 3.4 Computational speed and memory usage

**Figure 1:**
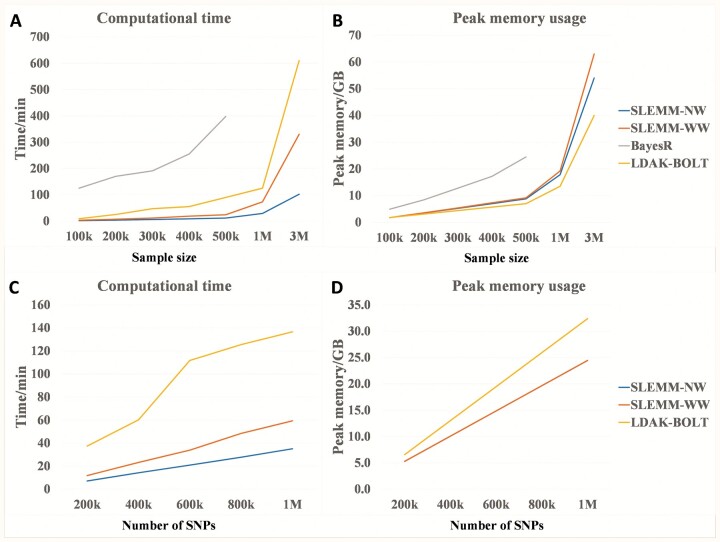
Computational performance of SLEMM, BayesR, and LDAK using simulation. (A and B) The number of SNPs is fixed to 50k. (C and D) The number of individuals is fixed to 100k. The line of SLEMM-NW overlaps that of SLEMM-WW in (D). LDAK computation did not include heritability estimation. The time of SLEMM-WW included two rounds of model fitting by REML

Simulation data were used to evaluate computing speed and peak memory usage. Ten threads were used for SLEMM, KAML, and LDAK, whereas four threads were used for BayesR. KAML and GCTA-NW could not handle large datasets. It took KAML 11 days to estimate SNP effects with 50k animals and 45k SNPs. SLEMM-NW is 0.5–2 times faster and uses slightly less memory than SLEMM-WW (SLEMM with window-based SNP weighting) (Fig. 1). SLEMM-WW is orders of magnitude faster than BayesR and 0.8-3 times faster than LDAK-BOLT (Fig. 1A and C). It is noteworthy that the LDAK-BOLT computation did not include *h*^2^ estimation. Both SLEMM and LDAK are much more memory-efficient than BayesR (Fig. 1B and D). LDAK-BOLT is slightly more memory-efficient than SLEMM when the number of SNPs is 50k (Fig. 1B). As the number of SNPs increases, SLEMM becomes more memory-efficient than LDAK-BOLT (Fig. 1D). In particular, SLEMM-WW used ∼330 min and 63 GB of peak memory for 3M individuals and 50k SNPs, and it used 59.3 min and 24.4 GB of peak memory for 100k individuals and 1M SNPs. The peak memory usage and computing time of SLEMM are largely proportional to sample size and sample size to the power of 1.5, respectively.

## 4 Discussion

It is challenging to use many genotyped and phenotyped individuals for genomic prediction. In this work, we present a new software tool, SLEMM, to address the challenge. SLEMM uses the Stochastic Lanczos REML and SNP weighting to effectively and accurately estimate SNP effects for large datasets. SLEMM is fast enough for million-scale genomic predictions (Fig. 1). In addition, SLEMM with window-based SNP weighting had an overall comparable predictive ability to BayesR for a variety of plant and livestock traits as shown in this work. BayesR has been demonstrated to outperform a variety of existing prediction methods (Zeng and Zhou 2017; Yin et al. 2020).

The choice of priors on SNP effects usually has a considerable impact on genomic prediction accuracy for traits underlain by a small number of large-effect QTLs (de los Campos et al. 2013). This was observed in our analysis; for example, SLEMM-WW, BayesR, and LDAK considerably improved predictions compared to SLEMM-NW for MY (milk) and MFP (milk fat percentage) in dairy bulls (Table 2). Such an improvement, however, nearly disappeared for the same traits in the much larger dairy cow data (Table 3). The result is in line with our expectation: priors become less influential as sample size increases.

The prediction accuracy of SNP weighting in SLEMM can be affected by the following settings: MAF weighting parameter, SNP window size, and the number of SNP weighting rounds. Our analysis on the two dairy datasets showed that the optimal MAF weighting parameter was consistent across traits and robust for each trait. We also found that genomic prediction benefited little from MAF-optimized SNP weighting compared to non-weighted SLEMM (Tables 2 and 3). SLEMM generally worked well with a window size of 20 SNPs for medium-density SNP chip data, though tuning it with cross-validation might improve predictive ability. A larger SNP window size may work better for a trait not underlain by large-effect QTLs. The number of iterative SNP weighting rounds can be fixed to one in SLEMM for all traits, because increasing it does not help even for a trait underlain by large-effect QTLs (Supplementary Fig. S18). Collectively, SLEMM requires little tuning on the three settings in most cases.

There have been several methods for leveraging functional annotations to improve genomic predictions, e.g. LDpred-funct (Márquez-Luna et al. 2021), LDAK (Zhang et al. 2021), and GMRM (Orliac et al. 2022). It is feasible to do so in SLEMM similar to LDAK and LDpred-funct: first, a software tool like S-LDSC (Finucane et al. 2015) is used to get functionally informed variance estimates for individual SNPs; second, SLEMM takes these variance estimates as input to weight SNPs. Further research is needed to develop this approach for livestock and plant data.

## Supplementary Material

btad127_Supplementary_Data

## Data Availability

Table 1 lists all the public datasets used in this study. The large dairy cow dataset is managed by CDCB. A request to CDCB that is necessary for getting data on research may be sent to João Dürr, CDCB Chief Executive Officer (joao.durr@cdcb.us).
