# Supplementary material for: SLEMM: million-scale genomic predictions with window-based SNP weighting
Source: Bioinformatics. 2023 Mar 10;39(3):btad127. doi: 10.1093/bioinformatics/btad127 (PMC10039786; doi:10.1093/bioinformatics/btad127)
Supplement: btad127_Supplementary_Data [file btad127_supplementary_data.zip › SLEMM-Supplementary-Methods.pdf]

**SLEMM Supplementary Methods**

**1 Statistical model**

SLEMM fits the following linear mixed model:

$$\begin{aligned} \mathbf{y} &= \mathbf{X}\boldsymbol{\beta} + \mathbf{Z}\boldsymbol{\alpha} + \mathbf{e} \\ \boldsymbol{\alpha} &\sim N(0, \mathbf{W}\sigma_\alpha^2) \,, \\ \mathbf{e} &\sim N(0, \mathbf{R}\sigma_e^2) \end{aligned} \quad (1)$$

where **y** is an *n*-by-1 vector of phenotypes for a quantitative trait, **β** is a *c*-by-1 vector of fixed effects, **X** is the *n*-by-*c* design matrix of full rank for **β**, **α** is an *m*-by-1 vector of SNP effects with diagonal covariance matrix $\mathbf{W}\sigma_\alpha^2$, **Z** is an *n*-by-*m* matrix of standardized genotypes, and **e** is an *n*-by-1 vector of residuals with diagonal covariance matrix $\mathbf{R}\sigma_e^2$. **R** is often equal to an identity matrix; however, when using deregressed estimated breeding values as **y** in a farm animal population, we need to precompute **R** to model their reliability [1]. Diagonal elements of **W** are weights with a mean of one for SNP effect variance; that is, $W_{jj}$ represents the relative contribution of SNP *j* to genetic variance. Optimal SNP weighting can improve genomic predictions.

The total variance-covariance matrix of model (1) is $\mathbf{V}_\sigma = \mathbf{Z}\mathbf{W}\mathbf{Z}^{\mathrm{T}}\sigma_\alpha^2 + \mathbf{R}\sigma_e^2$. We reparametrize it as $\mathbf{V}_\sigma = \mathbf{G}\sigma_g^2 + \mathbf{R}\sigma_e^2$, where $\mathbf{G} = \mathbf{Z}\mathbf{W}\mathbf{Z}^{\mathrm{T}}/m$ and $\sigma_g^2 = m\sigma_\alpha^2$. **G** is a genomic relationship matrix constructed with SNP weighting. $\mathbf{V}_\sigma$ can be rewritten as $\mathbf{V}_\sigma = \mathbf{H}_\tau\sigma_g^2$, in which $\mathbf{H}_\tau = \mathbf{G} + \mathbf{R}\tau$ and $\tau = \sigma_e^2/\sigma_g^2$.

**2 Log-likelihood**

Let **A** denote a matrix whose columns form an orthonormal basis for $\mathrm{span}(\mathbf{X})^{\perp}$ so that $\mathbf{A}^{\mathrm{T}}\mathbf{X} = \mathbf{0}$ and $\mathbf{A}^{\mathrm{T}}\mathbf{A} = \mathbf{I}$. We thus get the following model for restricted or residual maximum likelihood (REML):

$$\mathbf{A}^{\mathrm{T}}\mathbf{y} \sim N(0, \mathbf{A}^{\mathrm{T}}\mathbf{H}_\tau\mathbf{A}\sigma_g^2) \,,$$

which removes fixed effects compared to model (1). Its REML log-likelihood function is

$$\ell_{\text{REML}}(\sigma_g^2,\tau) = -\frac{n-c}{2}\log\left(2\pi\sigma_g^2\right) - \frac{1}{2}\log\left|\mathbf{A}^{\text{T}}\mathbf{H}_\tau\mathbf{A}\right| - \frac{1}{2}\mathbf{y}^{\text{T}}\mathbf{A}\left(\mathbf{A}^{\text{T}}\mathbf{H}_\tau\mathbf{A}\sigma_g^2\right)^{-1}\mathbf{A}^{\text{T}}\mathbf{y},$$

which is equivalent to

$$\ell_{\text{REML}}(\sigma_g^2,\tau) = -\frac{n-c}{2}\log\left(2\pi\sigma_g^2\right) + \frac{1}{2}\log\left|\mathbf{X}^{\text{T}}\mathbf{X}\right| - \frac{1}{2}\log\left|\mathbf{H}_\tau\right| - \frac{1}{2}\log\left|\mathbf{X}^{\text{T}}\mathbf{H}_\tau^{-1}\mathbf{X}\right| - \frac{1}{2}\mathbf{y}^{\text{T}}\mathbf{P}_\tau\mathbf{y}\sigma_g^{-2} \qquad (2)$$

where $\mathbf{P}_\tau = \mathbf{H}_\tau^{-1} - \mathbf{H}_\tau^{-1}\mathbf{X}(\mathbf{X}^{\text{T}}\mathbf{H}_\tau^{-1}\mathbf{X})^{-1}\mathbf{X}^{\text{T}}\mathbf{H}_\tau^{-1}$. Derivation for this equivalence can be found in [2, 3]. It can be revealed by $\partial\ell_{\text{REML}}(\sigma_g^2,\tau)/\partial\sigma_g^2 = 0$ that REML must meet

$$\sigma_g^2 = \mathbf{y}^{\text{T}}\mathbf{P}_\tau\mathbf{y}/(n-c). \qquad (3)$$

Taking equation (3) to (2), we parametrize the REML log-likelihood function with only $\tau$:

$$\begin{aligned}\ell_{\text{REML}}(\tau) = -\frac{n-c}{2} - \frac{n-c}{2}\log\left(\frac{2\pi}{n-c}\right) + \frac{1}{2}\log\left|\mathbf{X}^{\text{T}}\mathbf{X}\right| \\ -\frac{1}{2}\log\left|\mathbf{H}_\tau\right| - \frac{1}{2}\log\left|\mathbf{X}^{\text{T}}\mathbf{H}_\tau^{-1}\mathbf{X}\right| - \frac{n-c}{2}\log\left(\mathbf{y}^{\text{T}}\mathbf{P}_\tau\mathbf{y}\right)\end{aligned}. \qquad (4)$$

We can compute $\tau$'s REML estimate by optimizing (4) and then use (3) to estimate $\sigma_g^2$. In addition, we can reparametrize (2) regarding $\sigma_g^2$ and $\sigma_e^2$ as

$$\ell_{\text{REML}}(\sigma_g^2,\sigma_e^2) = -\frac{n-c}{2}\log\left(2\pi\right) + \frac{1}{2}\log\left|\mathbf{X}^{\text{T}}\mathbf{X}\right| - \frac{1}{2}\log\left|\mathbf{V}_\sigma\right| - \frac{1}{2}\log\left|\mathbf{X}^{\text{T}}\mathbf{V}_\sigma^{-1}\mathbf{X}\right| - \frac{1}{2}\mathbf{y}^{\text{T}}\mathbf{P}_\sigma\mathbf{y} \qquad (5)$$

where $\mathbf{P}_\sigma = \mathbf{P}_\tau\sigma_g^{-2}$. Note $\mathbf{P}_\sigma = \mathbf{P}_\sigma\mathbf{V}_\sigma\mathbf{P}_\sigma$ and $\mathbf{P}_\tau = \mathbf{P}_\tau\mathbf{H}_\tau\mathbf{P}_\tau$.

We next show a key equation for evaluating the REML log-likelihood functions. Let $\tilde{\mathbf{X}} = \mathbf{R}^{-1/2}\mathbf{X}$ and $\tilde{\mathbf{A}}$ denote a matrix whose columns form an orthonormal basis for $\text{span}(\tilde{\mathbf{X}})^\perp$ so that $\tilde{\mathbf{A}}^{\text{T}}\tilde{\mathbf{X}} = \mathbf{0}$ and $\tilde{\mathbf{A}}^{\text{T}}\tilde{\mathbf{A}} = \mathbf{I}$. Following the Proposition 1 in [3], we can get

$$\tilde{\mathbf{H}}_\tau^{-1} - \tilde{\mathbf{H}}_\tau^{-1}\tilde{\mathbf{X}}(\tilde{\mathbf{X}}^{\text{T}}\tilde{\mathbf{H}}_\tau^{-1}\tilde{\mathbf{X}})^{-1}\tilde{\mathbf{X}}^{\text{T}}\tilde{\mathbf{H}}_\tau^{-1} = \tilde{\mathbf{A}}(\tilde{\mathbf{A}}^{\text{T}}\tilde{\mathbf{H}}_\tau\tilde{\mathbf{A}})^{-1}\tilde{\mathbf{A}}^{\text{T}}$$

where $\tilde{\mathbf{H}}_\tau = \mathbf{R}^{-1/2}\mathbf{H}_\tau\mathbf{R}^{-1/2} = \mathbf{R}^{-1/2}\mathbf{G}\mathbf{R}^{-1/2} + \tau\mathbf{I}$. It is easy to know that $\mathbf{C} = \tilde{\mathbf{A}}\tilde{\mathbf{A}}^{\text{T}}\mathbf{R}^{-1/2}\mathbf{G}\mathbf{R}^{-1/2}\tilde{\mathbf{A}}\tilde{\mathbf{A}}^{\text{T}} + \tau\mathbf{I}$ is positive-definite. We can further show

$$
\begin{aligned}
(\tilde{\mathbf{A}}^{\mathrm{T}}\tilde{\mathbf{H}}_{\tau}\tilde{\mathbf{A}})(\tilde{\mathbf{A}}^{\mathrm{T}}\mathbf{C}^{-1}\tilde{\mathbf{A}}) &= \tilde{\mathbf{A}}^{\mathrm{T}}\mathbf{R}^{-1/2}\mathbf{G}\mathbf{R}^{-1/2}\tilde{\mathbf{A}}\tilde{\mathbf{A}}^{\mathrm{T}}\mathbf{C}^{-1}\tilde{\mathbf{A}} + \tau\tilde{\mathbf{A}}^{\mathrm{T}}\mathbf{C}^{-1}\tilde{\mathbf{A}} \\
&= \tilde{\mathbf{A}}^{\mathrm{T}}\tilde{\mathbf{A}}\tilde{\mathbf{A}}^{\mathrm{T}}\mathbf{R}^{-1/2}\mathbf{G}\mathbf{R}^{-1/2}\tilde{\mathbf{A}}\tilde{\mathbf{A}}^{\mathrm{T}}\mathbf{C}^{-1}\tilde{\mathbf{A}} + \tau\tilde{\mathbf{A}}^{\mathrm{T}}\mathbf{C}^{-1}\tilde{\mathbf{A}} \\
&= \tilde{\mathbf{A}}^{\mathrm{T}}(\tilde{\mathbf{A}}\tilde{\mathbf{A}}^{\mathrm{T}}\mathbf{R}^{-1/2}\mathbf{G}\mathbf{R}^{-1/2}\tilde{\mathbf{A}}\tilde{\mathbf{A}}^{\mathrm{T}} + \tau\mathbf{I})\mathbf{C}^{-1}\tilde{\mathbf{A}} \\
&= \mathbf{I}
\end{aligned}
$$

and similarly, $(\tilde{\mathbf{A}}^{\mathrm{T}}\mathbf{C}^{-1}\tilde{\mathbf{A}})(\tilde{\mathbf{A}}^{\mathrm{T}}\tilde{\mathbf{H}}_{\tau}\tilde{\mathbf{A}}) = \mathbf{I}$. Therefore, $(\tilde{\mathbf{A}}^{\mathrm{T}}\tilde{\mathbf{H}}_{\tau}\tilde{\mathbf{A}})^{-1} = \tilde{\mathbf{A}}^{\mathrm{T}}\mathbf{C}^{-1}\tilde{\mathbf{A}}$. It is then straightforward to show

$$
\begin{aligned}
\mathbf{P}_{\tau} &= \mathbf{H}_{\tau}^{-1} - \mathbf{H}_{\tau}^{-1}\mathbf{X}(\mathbf{X}^{\mathrm{T}}\mathbf{H}_{\tau}^{-1}\mathbf{X})^{-1}\mathbf{X}^{\mathrm{T}}\mathbf{H}_{\tau}^{-1} \\
&= \mathbf{R}^{-1/2}\left[\tilde{\mathbf{H}}_{\tau}^{-1} - \tilde{\mathbf{H}}_{\tau}^{-1}\tilde{\mathbf{X}}(\tilde{\mathbf{X}}^{\mathrm{T}}\tilde{\mathbf{H}}_{\tau}^{-1}\tilde{\mathbf{X}})^{-1}\tilde{\mathbf{X}}^{\mathrm{T}}\tilde{\mathbf{H}}_{\tau}^{-1}\right]\mathbf{R}^{-1/2} \\
&= \mathbf{R}^{-1/2}\tilde{\mathbf{A}}(\tilde{\mathbf{A}}^{\mathrm{T}}\tilde{\mathbf{H}}_{\tau}\tilde{\mathbf{A}})^{-1}\tilde{\mathbf{A}}^{\mathrm{T}}\mathbf{R}^{-1/2} \\
&= \mathbf{R}^{-1/2}\tilde{\mathbf{A}}\tilde{\mathbf{A}}^{\mathrm{T}}\mathbf{C}^{-1}\tilde{\mathbf{A}}\tilde{\mathbf{A}}^{\mathrm{T}}\mathbf{R}^{-1/2}
\end{aligned}
.
$$

Let $\mathbf{Q}$ denote the $Q$ matrix from the thin QR decomposition of $\tilde{\mathbf{X}}$. We can easily get $\tilde{\mathbf{A}}\tilde{\mathbf{A}}^{\mathrm{T}} = \mathbf{I} - \mathbf{Q}\mathbf{Q}^{\mathrm{T}}$, so

$$
\mathbf{P}_{\tau} = \mathbf{R}^{-1/2}(\mathbf{I}-\mathbf{Q}\mathbf{Q}^{\mathrm{T}})[(\mathbf{I}-\mathbf{Q}\mathbf{Q}^{\mathrm{T}})\mathbf{R}^{-1/2}\mathbf{G}\mathbf{R}^{-1/2}(\mathbf{I}-\mathbf{Q}\mathbf{Q}^{\mathrm{T}}) + \tau\mathbf{I}]^{-1}(\mathbf{I}-\mathbf{Q}\mathbf{Q}^{\mathrm{T}})\mathbf{R}^{-1/2}. \quad (6)
$$

## 3 Numerical optimization

We here provide an overview of the Lanczos algorithm in the context of REML, focusing on the framework but skipping the long derivations. Border and Becker [4] have presented most technical details on the method. We particularly address those not covered by their study.

### 3.1 Overview of the Lanczos algorithm

The Lanczos algorithm is an iterative Krylov subspace method devised by Lanczos for solving eigenvalue problems [5]. Though it works for any Hermitian matrix, we here consider only real symmetric positive-definite (SPD) matrices. For a real SPD matrix $\mathbf{A}$ of size $n$ by $n$ and a nonzero vector $\mathbf{b}$ of size $n$, the Lanczos algorithm generates the following recurrence decomposition:

$$
\mathbf{A}\mathbf{U}_m = \mathbf{U}_m\mathbf{T}_m + \beta_{m+1}\mathbf{u}_{m+1}\mathbf{e}_m^{\mathrm{T}}, \quad (7)
$$

where $\mathbf{U}_m$ is an $n$-by-$m$ matrix whose columns (denoted by $\mathbf{u}_1,\ldots,\mathbf{u}_m$) form an orthonormal basis for the Krylov subspace $\mathrm{span}\{\mathbf{b}, \mathbf{A}\mathbf{b}, \cdots, \mathbf{A}^{m-1}\mathbf{b}\}$, $\mathbf{T}_m$ is an $m$-by-$m$ symmetric tridiagonal matrix called the Jacobi

matrix with $\alpha_1,\ldots,\alpha_m$ on the diagonal and $\beta_2,\ldots,\beta_m$ on the subdiagonal and superdiagonal, and $\mathbf{e}_m$ is the $m$th column of the $m$-by-$m$ identity matrix. Note that $\mathbf{A}$, $\alpha$, $\beta$, and $\mathbf{e}$ all have a different denotation in the REML log-likelihood function or model (1) described above. We have $m \ll n$ in genomic REML.

Paige analyzed four variants of the Lanczos recursion procedure for computing $\mathbf{U}_m$ and $\mathbf{T}_m$ and denoted them by A(1, 6), A(1, 7), A(2, 6), and A(2, 7) [6]. A(2, 7) has been shown to be the most numerically stable [7], so we use it in our implementation. Border and Becker [4] used A(1, 7). The A(2, 7) recursion is given below:

$$\begin{aligned}
&\mathbf{u}_0 = \mathbf{0},\ \mathbf{u}_1 = \mathbf{b}/\|\mathbf{b}\|,\ \beta_1 = 0\\
&\text{for } k = 1,\cdots,m\\
&\qquad \mathbf{w}_k = \mathbf{A}\mathbf{u}_k - \beta_k \mathbf{u}_{k-1}\\
&\qquad \alpha_k = \mathbf{w}_k^{\mathrm{T}}\mathbf{u}_k\\
&\qquad \mathbf{u}_{k+1} = \mathbf{w}_k - \alpha_k \mathbf{u}_k\\
&\qquad \beta_{k+1} = \|\mathbf{u}_{k+1}\|\\
&\qquad \mathbf{u}_{k+1} = \mathbf{u}_{k+1}/\beta_{k+1}
\end{aligned} \quad . \tag{8}$$

Note that $\mathbf{A}$ is only involved in a matrix-vector product in (8), and $\mathbf{A}$ does not need to be explicitly computed. We can readily compute the approximate solution of $\mathbf{Ax} = \mathbf{b}$ with the Lanczos decomposition [8, 9]:

$$\tilde{\mathbf{x}} = \mathbf{U}_m \tilde{\mathbf{z}} \ \text{ with } \ \tilde{\mathbf{z}} = \|\mathbf{b}\| \mathbf{T}_m^{-1} \mathbf{e}_1, \tag{9}$$

where $\mathbf{U}_m$ and $\mathbf{T}_m$ are the same as defined in (7), and $\mathbf{e}_1$ is the first column of the $m$-by-$m$ identity matrix. The residual therefore is $\mathbf{r} = \mathbf{b} - \mathbf{A}\tilde{\mathbf{x}} = \mathbf{b} - (\mathbf{U}_m \mathbf{T}_m + \beta_{m+1}\mathbf{u}_{m+1}\mathbf{e}_m^{\mathrm{T}})\tilde{\mathbf{z}} = -\beta_{m+1}\mathbf{u}_{m+1}\mathbf{e}_m^{\mathbf{T}}\tilde{\mathbf{z}}$. Evaluating the residual is computationally cheap, and we can do it in each iteration of (8). Note that $m$ is not fixed. The stopping criterion of the Lanczos recursion is set as $\|\mathbf{r}\|/\|\mathbf{b}\|$ being smaller than a pre-specified tolerance (e.g., 5E-4). Border and Becker implemented a different but equivalent procedure (Algorithm 1 in [4]) for computing $\mathbf{r}$ and $\tilde{\mathbf{x}}$, by using the relationship between the Lanczos and conjugate gradient algorithms. As shown by [10, 11], the quantities obtained by the Lanczos recursion can be used to construct a complementary set of quantities for the conjugate gradient iterations, and vice versa. In addition, we can

apply (8) to multiple **b** vectors (denoted by $\mathbf{b}_1,\ldots,\mathbf{b}_L$) simultaneously to take advantage of fast BLAS (Basic Linear Algebra Subprograms) operations, with a conservative stopping criterion that $\|\mathbf{r}_l\|/\|\mathbf{b}_l\|$ (where $\mathbf{r}_l$ corresponds to $\mathbf{b}_l$) is smaller than a pre-specified tolerance for all $l$ from 1 to $L$ in an iteration [4].

The Lanczos algorithm can also be used to approximate log(det(**A**)) for a given SPD matrix **A**. The computation is based on $\log|\mathbf{A}| = \mathrm{tr}(\log(\mathbf{A}))$ and can be efficiently done by the Stochastic Lanczos Quadrature (SLQ) method [12]. Though the derivation on SLQ is tedious for non-specialists, SLQ is easy to implement (see Algorithm 1 in [12] and Algorithm 2 in [4]). In short, if we precompute the Lanczos decompositions of **A** with multiple normalized Rademacher random vectors $\mathbf{b}_l$ ($l$=1,…,$L$) (each element of $\mathbf{b}_l$ equal to either $-1/\|\mathbf{b}_l\|$ or $1/\|\mathbf{b}_l\|$ with equal probability), SLQ can estimate tr(log(**A**)) by the computation on just $\mathbf{T}_m^{(l)}$ ($l$=1,…,$L$) (where $\mathbf{T}_m^{(l)}$ corresponds to $\mathbf{b}_l$).

Krylov subspaces are shift-invariant. Given the Lanczos decomposition of SPD **A** as $\mathbf{A}\mathbf{U}_m = \mathbf{U}_m\mathbf{T}_m + \beta_{m+1}\mathbf{u}_{m+1}\mathbf{e}_m^{\mathrm{T}}$, it is easy to show that a shifted matrix of the form $\mathbf{A}_\delta = \mathbf{A} + \delta\mathbf{I}$ ($\delta$>0) has the following Lanczos decomposition

$$\mathbf{A}_\delta\mathbf{U}_m = \mathbf{U}_m(\mathbf{T}_m + \delta\mathbf{I}) + \beta_{m+1}\mathbf{u}_{m+1}\mathbf{e}_m^{\mathrm{T}}; \tag{10}$$

that is, the Lanczos decompositions of **A** and $\mathbf{A}_\delta$ differ only in the diagonal elements of their corresponding Jacobi matrices. Therefore, we can run the Lanczos recursion (8) for only **A** to Lanczos-decompose all shifted matrices $\mathbf{A}_\delta$ ($\delta$>0). In practice, the Lanczos recursion for **A** requires more iterations than $\mathbf{A}_\delta$ ($\delta$>0) to reach a pre-specified stopping criterion, so **A**'s Lanczos decomposition suffices $\mathbf{A}_\delta$'s.

### 3.2 Lanczos functions for REML

We construct three functions for using the Lanczos algorithm. The first one is based on the Lanczos recursion (8) and named LSeed(**A**, $\mathbf{B}=[\mathbf{b}_1\ldots\mathbf{b}_L]$), which takes **A** and **B** as input arguments and returns the **B**-based Lanczos decomposition of **A**. The second one is based on (9) and (10) and is named LSolve(LSeed(**A**, **B**), $\delta$≥0), which takes the output of LSeed(**A**, **B**) and $\delta$ as input arguments and returns the approximate solution of $(\mathbf{A}+\delta\mathbf{I})\mathbf{X}=\mathbf{B}$. The third function is SLQ(LSeed(**A**, $\mathbf{B}_R$), $\delta$≥0) and takes the

Jacobi matrix of LSeed(**A**, $\mathbf{B}_R$) and $\delta$ as input arguments and returns an approximate value of log(det(**A**+$\delta$**I**)), where each column of $\mathbf{B}_R$ is a normalized Rademacher random vector.

LSolve() and SLQ() are computationally cheap compared to LSeed(). The computation of LSeed(**A**, **b**) is dominated by the matrix-vector product $\mathbf{A}\mathbf{u}_k$ as in (8). Similarly, LSeed(**A**, **B**=[$\mathbf{b}_1 \ldots \mathbf{b}_L$]) is dominated by the computation of $\mathbf{A}\left[\mathbf{u}_k^{(1)} \quad \cdots \quad \mathbf{u}_k^{(L)}\right]$, in which $\mathbf{u}_k^{(l)}$ corresponds to $\mathbf{b}_l$ for $l$=1,…,$L$.

### 3.3 Stochastic Lanczos derivative-free REML

This method is based on the direct evaluation of the REML log-likelihood function (4). Our implementation is largely the same as Algorithm 3 in [4]. We first determine the minimum $\tau$ value (denoted by $\tau_0$) in $\mathbf{H}_\tau = \mathbf{G} + \mathbf{R}\tau$ by guesstimating an upper bound on SNP heritability. We then get the following Lanczos decompositions:

$$\begin{aligned} \mathrm{LS}_1 &= \mathrm{LSeed}(\mathbf{R}^{-1/2}\mathbf{ZWZ}^\mathrm{T}\mathbf{R}^{-1/2}/m + \mathbf{I}\tau_0, \mathbf{B}_R) \\ \mathrm{LS}_2 &= \mathrm{LSeed}(\mathbf{R}^{-1/2}\mathbf{ZWZ}^\mathrm{T}\mathbf{R}^{-1/2}/m + \mathbf{I}\tau_0, \mathbf{R}^{-1/2}\mathbf{X}) \\ \mathrm{LS}_3 &= \mathrm{LSeed}((\mathbf{I}-\mathbf{QQ}^\mathrm{T})\mathbf{R}^{-1/2}\mathbf{ZWZ}^\mathrm{T}\mathbf{R}^{-1/2}(\mathbf{I}-\mathbf{QQ}^\mathrm{T})/m + \mathbf{I}\tau_0, (\mathbf{I}-\mathbf{QQ}^\mathrm{T})\mathbf{R}^{-1/2}\mathbf{y}) \end{aligned},$$

where each column of $\mathbf{B}_R$ is a normalized Rademacher random vector, and other terms are defined the same as in model (1) and equation (6). Note that **A** in LSeed(**A**, **b**) has not been explicitly computed; for example, $\mathbf{A} = \mathbf{R}^{-1/2}\mathbf{ZWZ}^\mathrm{T}\mathbf{R}^{-1/2}/m + \mathbf{I}\tau_0$ in $\mathrm{LS}_1$, and the computation of $\mathbf{A}\mathbf{u}_k$ as in the Lanczos recursion (8) is reduced to $\mathbf{A}\mathbf{u}_k = \mathbf{R}^{-1/2}\sum_j \mathbf{z}_j W_{jj}\left(\mathbf{z}_j^\mathrm{T}\mathbf{R}^{-1/2}\mathbf{u}_k\right)/m + \tau_0\mathbf{u}_k$ in which $\mathbf{z}_j$ denotes the $j$th column of **Z** and $W_{jj}$ is the $j$th diagonal element of **W**.

The REML log-likelihood function (4) is therefore equal to

$$\begin{aligned} \ell_{\mathrm{REML}}(\tau) = \mathrm{Constant} - \frac{1}{2}\mathrm{SLQ}\left(\mathrm{LS}_1, \tau-\tau_0\right) - \frac{1}{2}\log\left|\mathbf{X}^\mathrm{T}\mathbf{R}^{-1/2}\left(\mathrm{LSolve}\left(\mathrm{LS}_2, \tau-\tau_0\right)\right)\right| \\ -\frac{n-c}{2}\log\left(\mathbf{y}^\mathrm{T}\mathbf{R}^{-1/2}(\mathbf{I}-\mathbf{QQ}^\mathrm{T})\left(\mathrm{LSolve}\left(\mathrm{LS}_3, \tau-\tau_0\right)\right)\right) \end{aligned},$$

which is computationally cheap to evaluate. Note that equation (6) has been used to show

$$\mathbf{P}_\tau\mathbf{y} = \mathbf{R}^{-1/2}(\mathbf{I}-\mathbf{QQ}^\mathrm{T})\left(\mathrm{LSolve}\left(\mathrm{LS}_3, \tau-\tau_0\right)\right). \quad (11)$$

We implement the golden-section search for finding the REML estimate of $\tau$ in [$\tau_0$, 9999]. REML estimates of variance components can be computed by equation (3).

### 3.4 Lanczos first-order Monte Carlo REML

Using the matrix calculus rules, particularly

$$\begin{aligned}\partial\left(\log\left|\mathbf{V}\right|\right) &= \mathrm{tr}\left(\mathbf{V}^{-1}\partial\mathbf{V}\right)\\ \partial\left(\mathbf{V}^{-1}\right) &= -\mathbf{V}^{-1}\partial\left(\mathbf{V}\right)\mathbf{V}^{-1}\end{aligned},$$

we can show

$$\begin{aligned}\frac{\partial\left(\log\left|\mathbf{V}_\sigma\right|+\log\left|\mathbf{X}^{\mathrm{T}}\mathbf{V}_\sigma^{-1}\mathbf{X}\right|\right)}{\partial\sigma_g^2} &= \mathrm{tr}(\mathbf{V}_\sigma^{-1}\mathbf{G})-\mathrm{tr}((\mathbf{X}^{\mathrm{T}}\mathbf{V}_\sigma^{-1}\mathbf{X})^{-1}\mathbf{X}^{\mathrm{T}}\mathbf{V}_\sigma^{-1}\mathbf{G}\mathbf{V}_\sigma^{-1}\mathbf{X})\\ &= \mathrm{tr}(\mathbf{V}_\sigma^{-1}\mathbf{G})-\mathrm{tr}(\mathbf{V}_\sigma^{-1}\mathbf{X}(\mathbf{X}^{\mathrm{T}}\mathbf{V}_\sigma^{-1}\mathbf{X})^{-1}\mathbf{X}^{\mathrm{T}}\mathbf{V}_\sigma^{-1}\mathbf{G})\\ &= \mathrm{tr}(\mathbf{P}_\sigma\mathbf{G})\end{aligned}$$

and

$$\begin{aligned}\frac{\partial\mathbf{P}_\sigma}{\partial\sigma_g^2} &= \frac{\partial(\mathbf{V}_\sigma^{-1}-\mathbf{V}_\sigma^{-1}\mathbf{X}(\mathbf{X}^{\mathrm{T}}\mathbf{V}_\sigma^{-1}\mathbf{X})^{-1}\mathbf{X}^{\mathrm{T}}\mathbf{V}_\sigma^{-1})}{\partial\sigma_g^2}\\ &= -\mathbf{V}_\sigma^{-1}\mathbf{G}\mathbf{V}_\sigma^{-1}+\mathbf{V}_\sigma^{-1}\mathbf{G}\mathbf{V}_\sigma^{-1}\mathbf{X}(\mathbf{X}^{\mathrm{T}}\mathbf{V}_\sigma^{-1}\mathbf{X})^{-1}\mathbf{X}^{\mathrm{T}}\mathbf{V}_\sigma^{-1}+\mathbf{V}_\sigma^{-1}\mathbf{X}(\mathbf{X}^{\mathrm{T}}\mathbf{V}_\sigma^{-1}\mathbf{X})^{-1}\mathbf{X}^{\mathrm{T}}\mathbf{V}_\sigma^{-1}\mathbf{G}\mathbf{V}_\sigma^{-1}\\ &\quad -\mathbf{V}_\sigma^{-1}\mathbf{X}(\mathbf{X}^{\mathrm{T}}\mathbf{V}_\sigma^{-1}\mathbf{X})^{-1}\mathbf{X}^{\mathrm{T}}\mathbf{V}_\sigma^{-1}\mathbf{G}\mathbf{V}_\sigma^{-1}\mathbf{X}(\mathbf{X}^{\mathrm{T}}\mathbf{V}_\sigma^{-1}\mathbf{X})^{-1}\mathbf{X}^{\mathrm{T}}\mathbf{V}_\sigma^{-1}\\ &= -(\mathbf{V}_\sigma^{-1}-\mathbf{V}_\sigma^{-1}\mathbf{X}(\mathbf{X}^{\mathrm{T}}\mathbf{V}_\sigma^{-1}\mathbf{X})^{-1}\mathbf{X}^{\mathrm{T}}\mathbf{V}_\sigma^{-1})\mathbf{G}(\mathbf{V}_\sigma^{-1}-\mathbf{V}_\sigma^{-1}\mathbf{X}(\mathbf{X}^{\mathrm{T}}\mathbf{V}_\sigma^{-1}\mathbf{X})^{-1}\mathbf{X}^{\mathrm{T}}\mathbf{V}_\sigma^{-1})\\ &= -\mathbf{P}_\sigma\mathbf{G}\mathbf{P}_\sigma\end{aligned}.$$

Similarly, $\partial\left(\log\left|\mathbf{V}_\sigma\right|+\log\left|\mathbf{X}^{\mathrm{T}}\mathbf{V}_\sigma^{-1}\mathbf{X}\right|\right)\big/\partial\sigma_e^2 = \mathrm{tr}(\mathbf{P}_\sigma\mathbf{R})$ and $\partial\mathbf{P}_\sigma\big/\partial\sigma_e^2 = -\mathbf{P}_\sigma\mathbf{R}\mathbf{P}_\sigma$. It is then straightforward to get the first derivatives for function (5):

$$\begin{aligned}\frac{\ell_{\mathrm{REML}}(\sigma_g^2,\sigma_e^2)}{\partial\sigma_g^2} &= -\frac{1}{2}\mathrm{tr}\left(\mathbf{P}_\sigma\mathbf{G}\right)+\frac{1}{2}\mathbf{y}^{\mathrm{T}}\mathbf{P}_\sigma\mathbf{G}\mathbf{P}_\sigma\mathbf{y}\\ \frac{\ell_{\mathrm{REML}}(\sigma_g^2,\sigma_e^2)}{\partial\sigma_e^2} &= -\frac{1}{2}\mathrm{tr}\left(\mathbf{P}_\sigma\mathbf{R}\right)+\frac{1}{2}\mathbf{y}^{\mathrm{T}}\mathbf{P}_\sigma\mathbf{R}\mathbf{P}_\sigma\mathbf{y}\end{aligned}.$$

The first-order REML conditions are

$$\begin{aligned}\mathbf{y}^{\mathrm{T}}\mathbf{P}_\sigma\mathbf{G}\mathbf{P}_\sigma\mathbf{y} &= \mathrm{tr}\left(\mathbf{P}_\sigma\mathbf{G}\right)\\ \mathbf{y}^{\mathrm{T}}\mathbf{P}_\sigma\mathbf{R}\mathbf{P}_\sigma\mathbf{y} &= \mathrm{tr}\left(\mathbf{P}_\sigma\mathbf{R}\right)\end{aligned}.$$

Following [4, 13, 14], we consider the function on $\tau$ below whose root meets the above REML conditions:

$$f_{\text{REML}}(\tau) = \log\left(\frac{\mathbf{y}^{\mathrm{T}}\mathbf{P}_{\sigma}\mathbf{G}\mathbf{P}_{\sigma}\mathbf{y}}{\mathbf{y}^{\mathrm{T}}\mathbf{P}_{\sigma}\mathbf{R}\mathbf{P}_{\sigma}\mathbf{y}}\right) - \log\left(\frac{\mathrm{tr}\left(\mathbf{P}_{\sigma}\mathbf{G}\right)}{\mathrm{tr}\left(\mathbf{P}_{\sigma}\mathbf{R}\right)}\right) = \log\left(\frac{\mathbf{y}^{\mathrm{T}}\mathbf{P}_{\tau}\mathbf{G}\mathbf{P}_{\tau}\mathbf{y}}{\mathbf{y}^{\mathrm{T}}\mathbf{P}_{\tau}\mathbf{R}\mathbf{P}_{\tau}\mathbf{y}}\right) - \log\left(\frac{\mathrm{tr}\left(\mathbf{P}_{\tau}\mathbf{G}\right)}{\mathrm{tr}\left(\mathbf{P}_{\tau}\mathbf{R}\right)}\right).$$

We apply stochastic trace estimators to tr($\mathbf{P}_\tau\mathbf{G}$) and tr($\mathbf{P}_\tau\mathbf{R}$) [15], rewriting the above function as

$$f_{\text{REML}}(\tau) = \log\left(\frac{\mathbf{y}^{\mathrm{T}}\mathbf{P}_{\tau}\mathbf{Z}\mathbf{W}\mathbf{Z}^{\mathrm{T}}\mathbf{P}_{\tau}\mathbf{y}}{\mathbf{y}^{\mathrm{T}}\mathbf{P}_{\tau}\mathbf{R}\mathbf{P}_{\tau}\mathbf{y}}\right) - \log\left(\frac{\mathrm{E}\left(\mathbf{y}_H^{\mathrm{T}}\mathbf{P}_{\tau}\mathbf{Z}\mathbf{W}\mathbf{Z}^{\mathrm{T}}\mathbf{P}_{\tau}\mathbf{y}_H\right)}{\mathrm{E}\left(\mathbf{y}_H^{\mathrm{T}}\mathbf{P}_{\tau}\mathbf{R}\mathbf{P}_{\tau}\mathbf{y}_H\right)}\right), \tag{12}$$

where $\mathbf{y}_H$ is a random vector such that $\mathrm{E}\left(\mathbf{y}_H\mathbf{y}_H^{\mathrm{T}}\right) = \mathbf{H}_\tau$. We can build $\mathbf{y}_H$ with $\mathbf{y}_H = \mathbf{Z}\mathbf{W}^{1/2}\mathbf{v}_1 / \sqrt{m} + \sqrt{\tau}\mathbf{R}^{1/2}\mathbf{v}_2$ in which $\mathbf{v}_1$ and $\mathbf{v}_2$ are random vectors of standard normal.

We get the following Lanczos decompositions:

$$\begin{aligned}\mathrm{LS}_4 &= \mathrm{LSeed}((\mathbf{I}-\mathbf{Q}\mathbf{Q}^{\mathrm{T}})\mathbf{R}^{-1/2}\mathbf{Z}\mathbf{W}\mathbf{Z}^{\mathrm{T}}\mathbf{R}^{-1/2}(\mathbf{I}-\mathbf{Q}\mathbf{Q}^{\mathrm{T}})/m + \mathbf{I}\tau_0, (\mathbf{I}-\mathbf{Q}\mathbf{Q}^{\mathrm{T}})\mathbf{R}^{-1/2}\mathbf{Z}\mathbf{W}^{1/2}\mathbf{v}_1/\sqrt{m}) \\ \mathrm{LS}_5 &= \mathrm{LSeed}((\mathbf{I}-\mathbf{Q}\mathbf{Q}^{\mathrm{T}})\mathbf{R}^{-1/2}\mathbf{Z}\mathbf{W}\mathbf{Z}^{\mathrm{T}}\mathbf{R}^{-1/2}(\mathbf{I}-\mathbf{Q}\mathbf{Q}^{\mathrm{T}})/m + \mathbf{I}\tau_0, (\mathbf{I}-\mathbf{Q}\mathbf{Q}^{\mathrm{T}})\mathbf{R}^{-1/2}\mathbf{R}^{1/2}\mathbf{v}_2)\end{aligned}.$$

It is then easy to show that

$$\mathbf{P}_\tau\mathbf{y}_H = \mathbf{R}^{-1/2}(\mathbf{I}-\mathbf{Q}\mathbf{Q}^{\mathrm{T}})\left(\mathrm{LSolve}(\mathrm{LS}_4, \tau-\tau_0) + \sqrt{\tau}\,\mathrm{LSolve}(\mathrm{LS}_5, \tau-\tau_0)\right). \tag{13}$$

So it becomes straightforward to evaluate (12) with equations (11) and (13). Following BOLT [13-15], we use the secant method for finding the root of (12). REML estimates of variance components are then computed by equation (3).

**4 Data**

Seven public datasets were included in this study. We also used a large dairy dataset from the U.S. Council on Dairy Cattle Breeding (CDCB).

**Cattle:** A dataset of 5,024 bulls from a German Holstein population [16] was used, including conventional estimated breeding values (EBVs) of milk fat percentage (MFP), milk yield (MY), and somatic cell score (SCS). These three traits represent distinct genetic architectures: MFP and MY are regulated by a few major QTLs and many small-effect loci, while SCS is underlain by many loci with small effects [16, 17]. All bulls were genotyped with the Illumina Bovine SNP50 Beadchip, with 42,551 SNPs remained for our analyses.

**Pig:** Two datasets were used. The first dataset consists of 2,785 Duroc boars with three traits: backfat thickness at 100 kg (BF, mm), loin muscle depth at 100 kg (LMD, mm), and total teat number (TTN) [18]. The phenotypes of all traits were pre-adjusted by contemporary group effects using a full GBLUP model. All three traits are highly polygenic, and a major QTL underlies TTN [18]. A subset of 258,662 SNPs with MAF >1%, LD $r^2$ <0.98, and a call rate >95% was retained from low-coverage sequencing. For our analyses, a total of 49,564 SNPs were randomly selected, with HWE *p*-value >1E-6 and MAF >5%. The second dataset consists of 4,260 pigs of four pure breeds (Duroc, Yorkshire, Landrace, and Pietrain) with two polygenic traits: average daily gain (ADG) and age adjusted to 100 kg [19]. Both traits were pre-adjusted by systematic environmental effects using a full GBLUP model. All animals were genotyped by the Illumina PorcineSNP50 Bead Chip. A total of 47,157 SNPs were retained for analyses after quality control using HWE *p*-value >1E-6 and MAF >5%.

**Chicken:** A total of 1,063 hens were available from [20], with phenotypes of the first egg weight (EW1), egg weight at 36 weeks of age (EW36), and egg weight at 56 weeks of age (EW56). All traits were found polygenic. All hens were genotyped by the Affymetrix 600K chicken SNP chip, and genotypes were quality-controlled using call rate >97%, MAF >1%, and HWE *p*-value >1E-6. For our analyses, a total of 44,430 SNPs were further randomly selected.

**Maize:** 1,868 and 1,631 inbred lines for sweet or starchy kernels (Sweet) and growing degree days (GDD), respectively, from [21] were used. Both traits were highly polygenic. All maize lines were genotyped by genotyping by sequencing. A total of 455,344 SNPs were retained after quality control using MAF >1%, HWE *p*-value >1E-6, and missing rate <5%. A subset of 45,426 SNPs was randomly selected for our analyses.

**Wheat:** The wheat data were obtained from the R package BLR [22], which contained 599 inbred lines with phenotypes of average grain yield (GY) evaluated in four different mega-environments (GY1, GY2, GY3, and GY4). All traits are polygenic. All lines were genotyped by Diversity Array Technology (DArT), and 1,279 markers were retained after quality control.

**Pine:** The pine tree data contained 861 individuals with two traits, diameter at breast height (DBH) and height at age of 6 years (HT), as described in [23]. Both traits are highly polygenic. All trees were genotyped using an Illumina Infinium assay, and a subset of 4,853 SNPs was retained and used in our analysis.

**CDCB dairy cow data:** As described by [24], the dataset consists of 294,079 Holstein cows with first-lactation phenotypes of five milk production traits (milk, fat, and protein [Pro] yields, and fat [FPC] and protein [PPC] percentages), three fertility traits (daughter pregnancy rate [DPR], cow conception rate [CCR], and heifer conception rate [HCR]), and somatic cell score (SCS). All traits are polygenic, and major QTLs were found for the milk production traits (Milk, Fat, Pro, FPC, and PPC) [17, 24]. All animals had genotypes of 60,671 SNPs imputed from the dairy genomic database at CDCB.

## References


1. VanRaden, P.M., *Efficient methods to compute genomic predictions.* Journal of dairy science, 2008. **91**(11): p. 4414-4423.
2. Harville, D.A., *Bayesian inference for variance components using only error contrasts.* Biometrika, 1974. **61**(2): p. 383-385.
3. LaMotte, L.R., *A direct derivation of the REML likelihood function.* Statistical Papers, 2007. **48**(2): p. 321-327.
4. Border, R. and S. Becker, *Stochastic Lanczos estimation of genomic variance components for linear mixed-effects models.* BMC bioinformatics, 2019. **20**(1): p. 1-16.
5. Lanczos, C., *An iteration method for the solution of the eigenvalue problem of linear differential and integral operators.* 1950.
6. Paige, C.C., *Computational variants of the Lanczos method for the eigenproblem.* IMA Journal of Applied Mathematics, 1972. **10**(3): p. 373-381.
7. *2. Lanczos Procedures, Real Symmetric Problems*, in *Lanczos Algorithms for Large Symmetric Eigenvalue Computations*. p. 32-75.
8. Paige, C.C. and M.A. Saunders, *Solution of sparse indefinite systems of linear equations.* SIAM journal on numerical analysis, 1975. **12**(4): p. 617-629.
9. Meerbergen, K., *The solution of parametrized symmetric linear systems.* SIAM journal on matrix analysis and applications, 2003. **24**(4): p. 1038-1059.
10. *4. Lanczos Procedures with No Reorthogonalization for Real Symmetric Problems*, in *Lanczos Algorithms for Large Symmetric Eigenvalue Computations*. p. 92-163.
11. Meurant, G. and Z. Strakoš, *The Lanczos and conjugate gradient algorithms in finite precision arithmetic.* Acta Numerica, 2006. **15**: p. 471-542.
12. Ubaru, S., J. Chen, and Y. Saad, *Fast estimation of tr(f(a)) via stochastic lanczos quadrature.* SIAM Journal on Matrix Analysis and Applications, 2017. **38**(4): p. 1075-1099.
13. Loh, P.-R., et al., *Efficient Bayesian mixed-model analysis increases association power in large cohorts.* Nature genetics, 2015. **47**(3): p. 284-290.
14. Loh, P.-R., et al., *Mixed-model association for biobank-scale datasets.* Nature genetics, 2018. **50**(7): p. 906-908.
15. Loh, P.-R., et al., *Contrasting genetic architectures of schizophrenia and other complex diseases using fast variance-components analysis.* Nature genetics, 2015. **47**(12): p. 1385-1392.
16. Zhang, Z., et al., *Accuracy of Whole-Genome Prediction Using a Genetic Architecture-Enhanced Variance-Covariance Matrix.* G3-Genes Genomes Genetics, 2015. **5**(4): p. 615-627.
17. Jiang, J., et al., *Functional annotation and Bayesian fine-mapping reveals candidate genes for important agronomic traits in Holstein bulls.* Commun Biol, 2019. **2**: p. 212.
18. Yang, R., et al., *Accelerated deciphering of the genetic architecture of agricultural economic traits in pigs using a low-coverage whole-genome sequencing strategy.* Gigascience, 2021. **10**(7).
19. Tang, Z., et al., *Genome-Wide Association Study Reveals Candidate Genes for Growth Relevant Traits in Pigs.* Front Genet, 2019. **10**: p. 302.
20. Liu, Z., et al., *Genome-Wide Association Analysis of Age-Dependent Egg Weights in Chickens.* Front Genet, 2018. **9**: p. 128.
21. Romay, M.C., et al., *Comprehensive genotyping of the USA national maize inbred seed bank.* Genome Biol, 2013. **14**(6): p. R55.
22. de Los Campos, G., et al., *Genome-enabled prediction using the BLR (Bayesian Linear Regression) R-package.* Methods Mol Biol, 2013. **1019**: p. 299-320.
23. Resende, M.F., Jr., et al., *Accuracy of genomic selection methods in a standard data set of loblolly pine (Pinus taeda L.).* Genetics, 2012. **190**(4): p. 1503-10.

24. Jiang, J., et al., *A Large-Scale Genome-Wide Association Study in U.S. Holstein Cattle.* Front Genet, 2019. **10**: p. 412.
