# Supplementary material for: SLEMM: million-scale genomic predictions with window-based SNP weighting
Source: Bioinformatics. 2023 Mar 10;39(3):btad127. doi: 10.1093/bioinformatics/btad127 (PMC10039786; doi:10.1093/bioinformatics/btad127)
Supplement: btad127_Supplementary_Data [file btad127_supplementary_data.zip › SLEMM-Supplementary-Figures.pdf]

Supplementary Figures

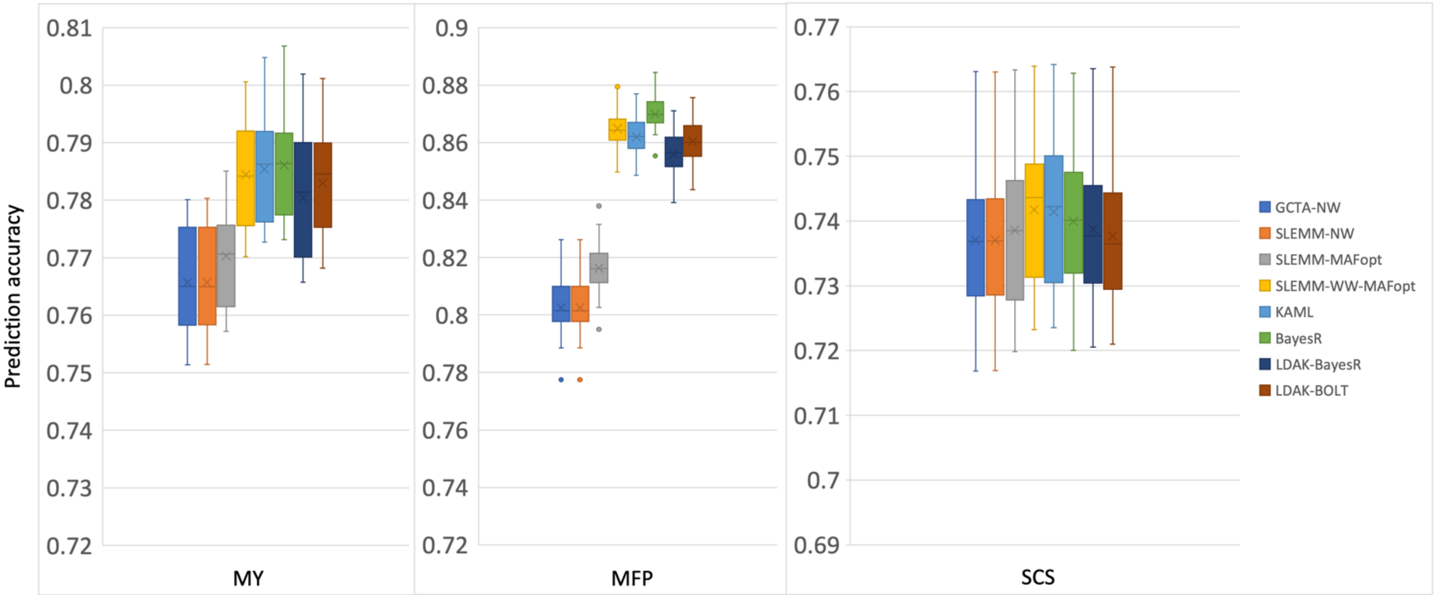

Figure S1. Predictive abilities of non-weighted GCTA (GCTA-NW), non-weighted SLEMM (SLEMM-NW), MAF-weight-optimized SLEMM (SLEMM-MAFopt), window-weighted and MAF-weight-optimized SLEMM (SLEMM-WW-MAFopt), KAML, BayesR, and LDAK using the dairy bull data

Data was randomly split into a training population (about 80% of individuals) and a validation population (about 20% of individuals) for 20 replicates. MY: milk yield; MFP: milk fat percentage; SCS: somatic cell score

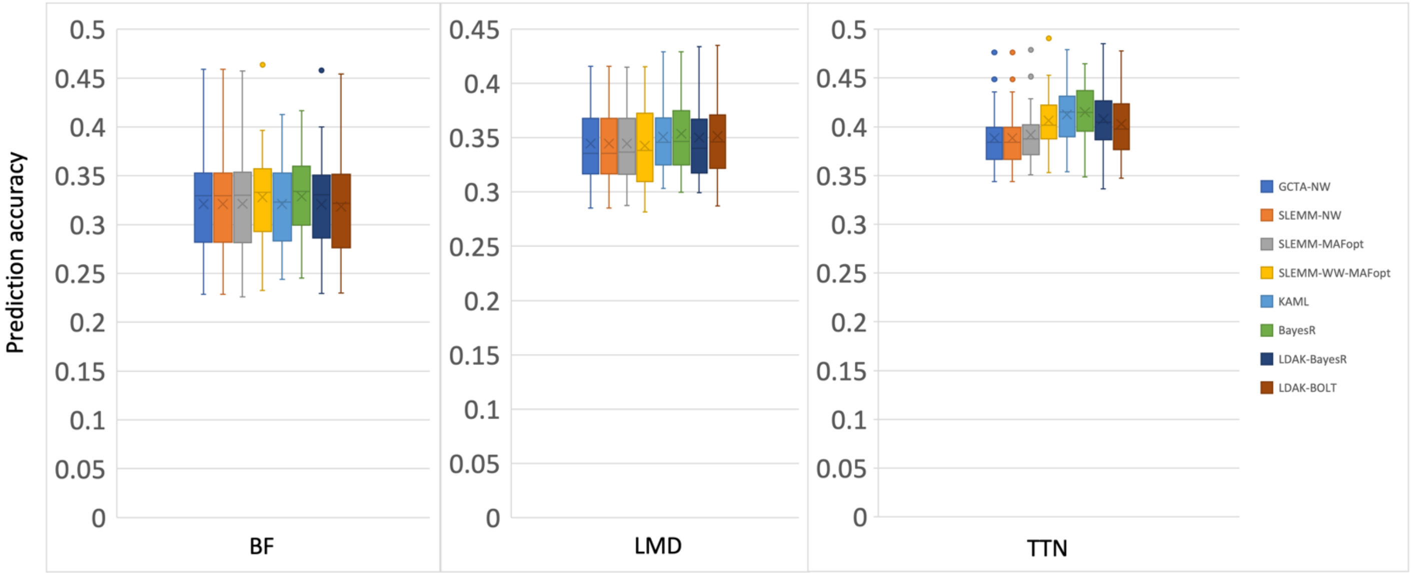

Figure S2. Predictive abilities of non-weighted GCTA (GCTA-NW), non-weighted SLEMM (SLEMM-NW), MAF-weight-optimized SLEMM (SLEMM-MAFopt), window-weighted and MAF-weight-optimized SLEMM (SLEMM-WW-MAFopt), KAML, BayesR, and LDAK in Duroc pigs

Data was randomly split into a training population (about 80% of individuals) and a validation population (about 20% of individuals) for 20 replicates. BF: backfat; LMD: loin muscle depth; TTN: total teat number

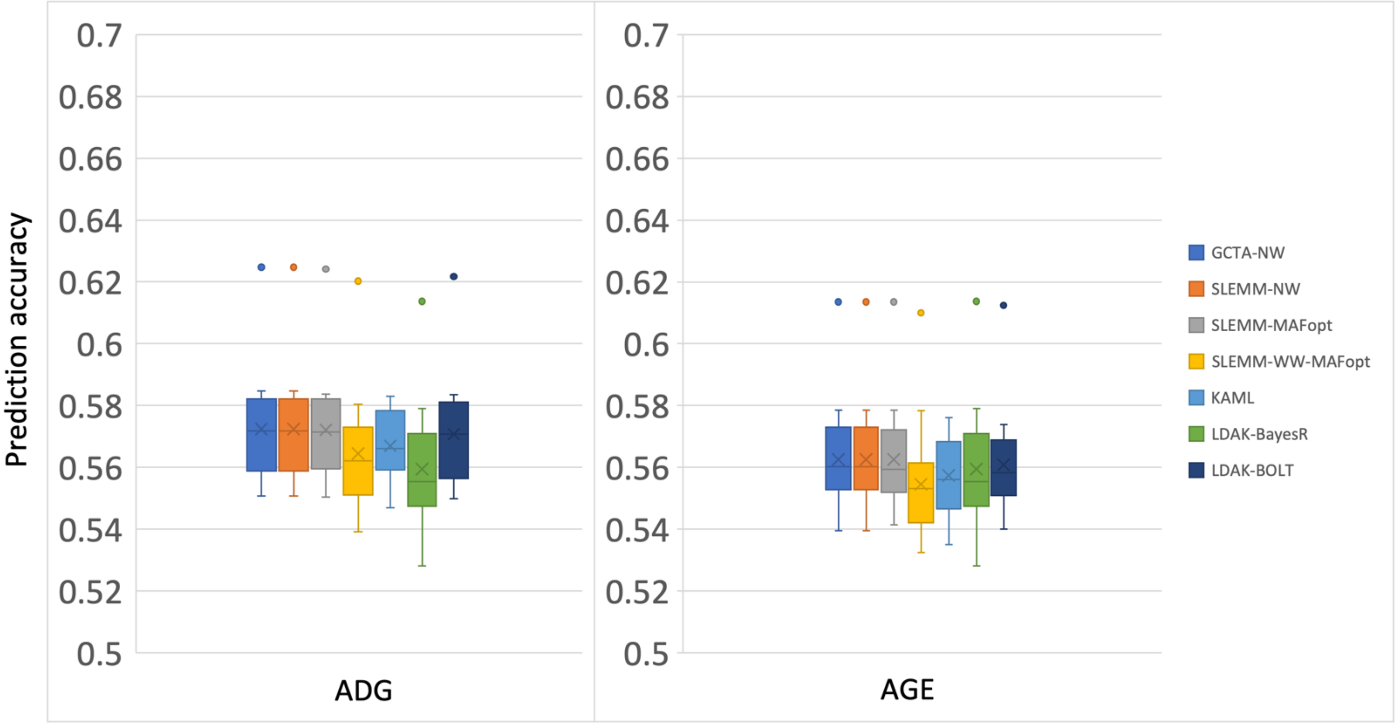

Figure S3. Predictive abilities of non-weighted GCTA (GCTA-NW), non-weighted SLEMM (SLEMM-NW), MAF-weight-optimized SLEMM (SLEMM-MAFopt), window-weighted and MAF-weight-optimized SLEMM (SLEMM-WW-MAFopt), KAML, BayesR, and LDAK in pigs from four different breeds

Data was randomly split into a training population (about 80% of individuals) and a validation population (about 20% of individuals) for 20 replicates. ADG: average daily gain; AGE: off test age.

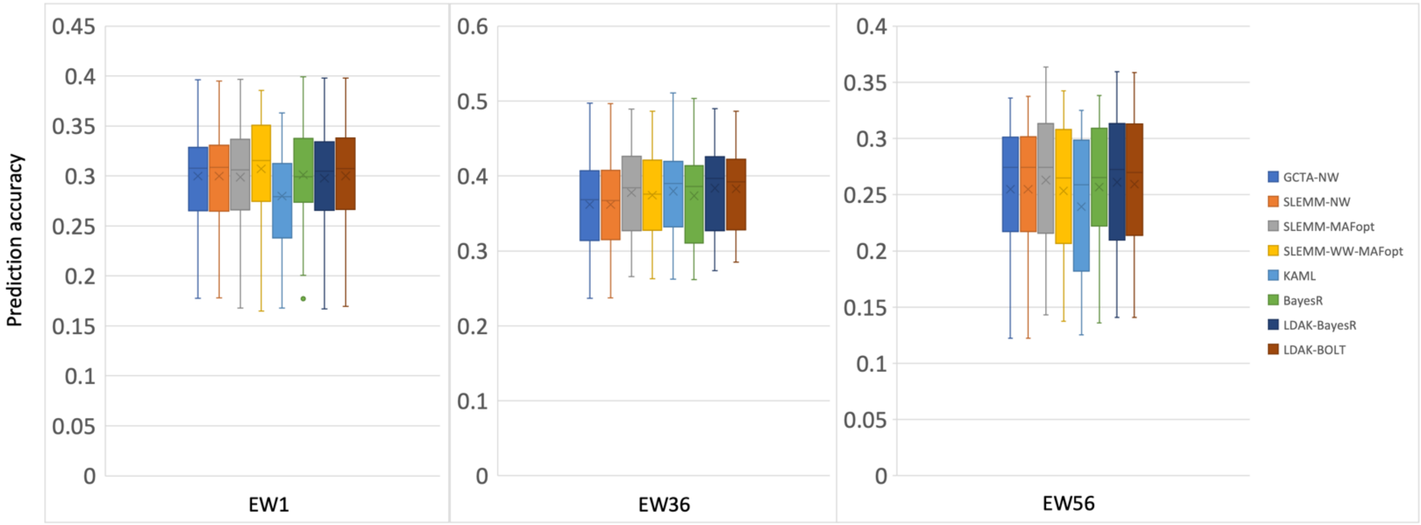

Figure S4. Predictive abilities of non-weighted GCTA (GCTA-NW), non-weighted SLEMM (SLEMM-NW), MAF-weight-optimized SLEMM (SLEMM-MAFopt), window-weighted and MAF-weight-optimized SLEMM (SLEMM-WW-MAFopt), KAML, BayesR, and LDAK in chicken

Data was randomly split into a training population (about 80% of individuals) and a validation population (about 20% of individuals) for 20 replicates. EW1: first egg weight; EW36: egg weight at 36 weeks old; EW56: egg weight at 56 weeks old

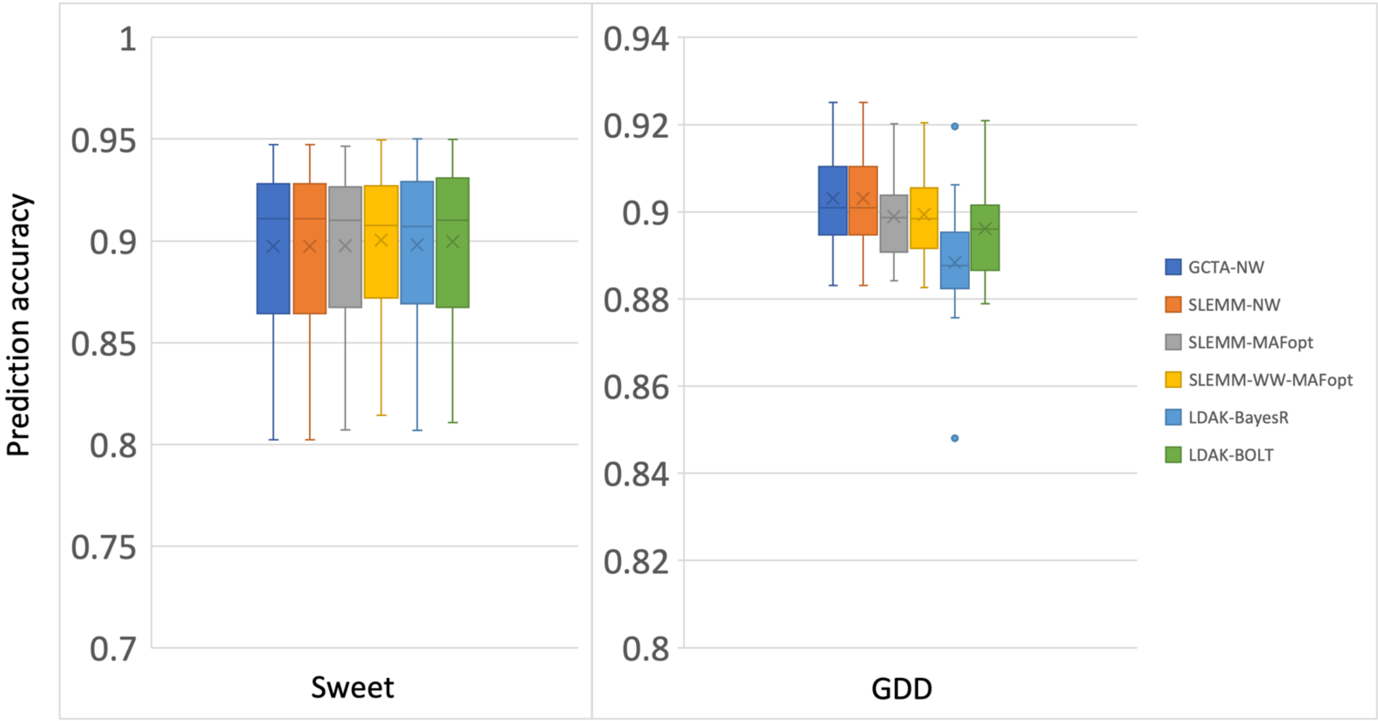

Figure S5. Predictive abilities of non-weighted GCTA (GCTA-NW), non-weighted SLEMM (SLEMM-NW), MAF-weight-optimized SLEMM (SLEMM-MAFopt), window-weighted and MAF-weight-optimized SLEMM (SLEMM-WW-MAFopt), and LDAK in maize

Data was randomly split into a training population (about 80% of individuals) and a validation population (about 20% of individuals) for 20 replicates. Sweet: kernel is sweet or starchy; GDD: growing degree days.

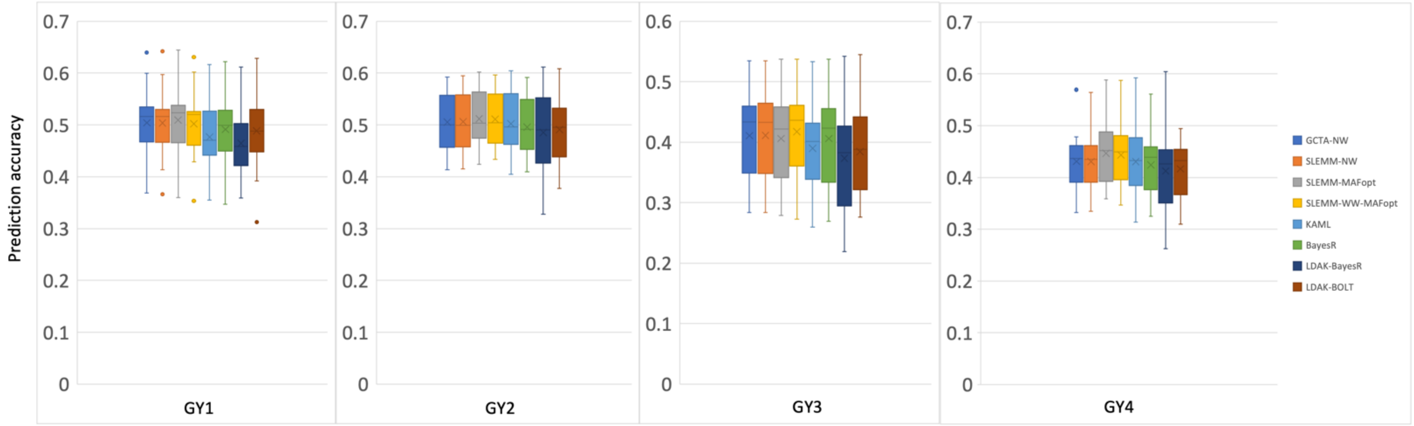

Figure S6. Predictive abilities of non-weighted GCTA (GCTA-NW), non-weighted SLEMM (SLEMM-NW), MAF-weight-optimized SLEMM (SLEMM-MAFopt), window-weighted and MAF-weight-optimized SLEMM (SLEMM-WW-MAFopt), KAML, BayesR, and LDAK in wheat

Data was randomly split into a training population (about 80% of individuals) and a validation population (about 20% of individuals) for 20 replicates. GY1: grain yield in environment 1; GY2: grain yield in environment 2; GY3: grain yield in environment 3; GY4: grain yield in environment 4.

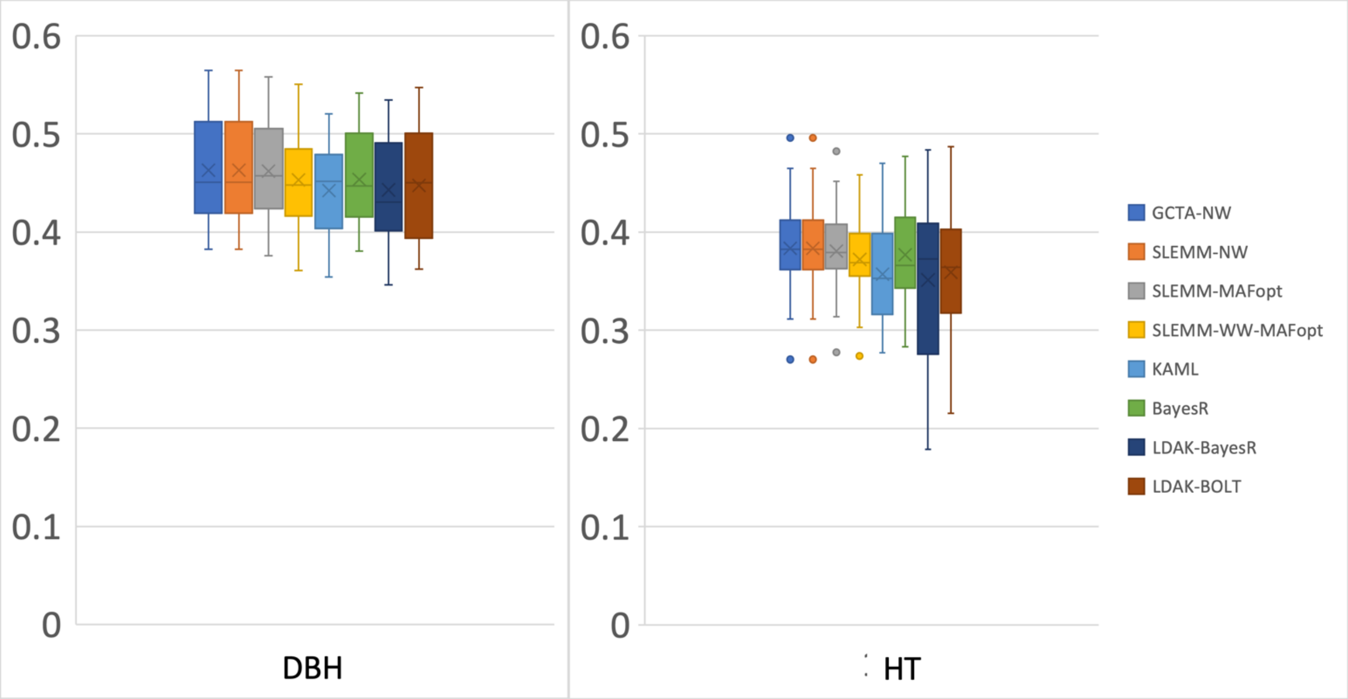

Figure S7. Predictive abilities of non-weighted GCTA (GCTA-NW), non-weighted SLEMM (SLEMM-NW), MAF-weight-optimized SLEMM (SLEMM-MAFopt), window-weighted and MAF-weight-optimized SLEMM (SLEMM-WW-MAFopt), KAML, BayesR, and LDAK in pine

Data was randomly split into a training population (about 80% of individuals) and a validation population (about 20% of individuals) for 20 replicates. DBH: stem diameter; HT: total stem height.

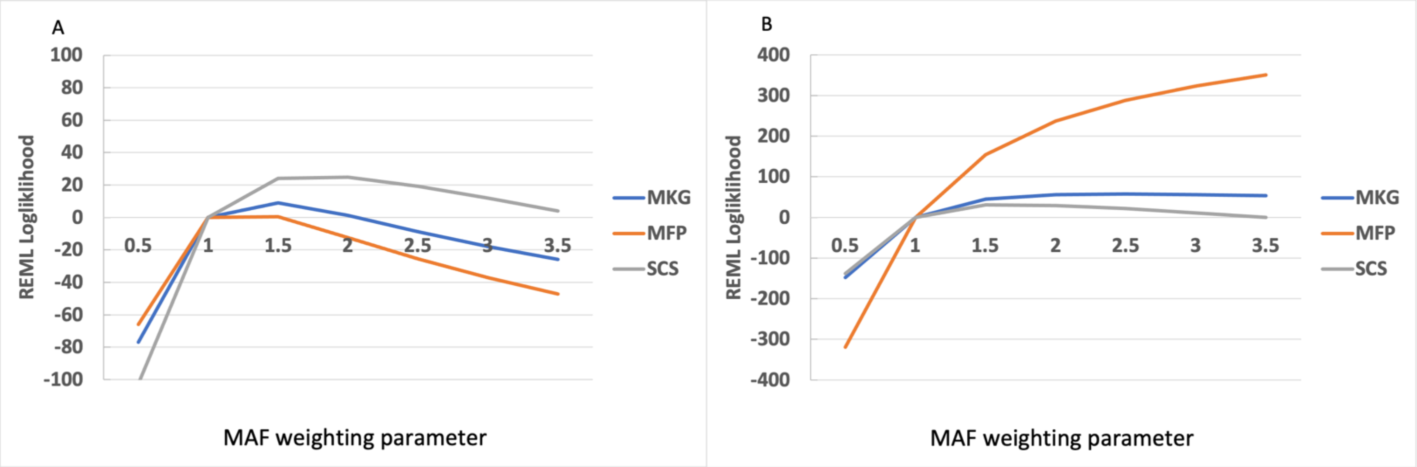

Figure S8. Relationship between GREML log-likelihood and the MAF weighting parameter using all individuals in the dairy bull data

A: LD-pruned, significant SNPs were fitted as fixed effects for MKG and MFP. B: No SNPs were fitted as fixed effects. MAF weighting parameter is a (=b), where a and b are the same as defined in equation (2) and a=b=1 denotes non-weighted empirical GBLUP. MKG: milk yield; MFP: milk fat percentage; SCS: somatic cell score. Log-likelihood was shifted so that the y-value for a=b=1 is zero for each trait.

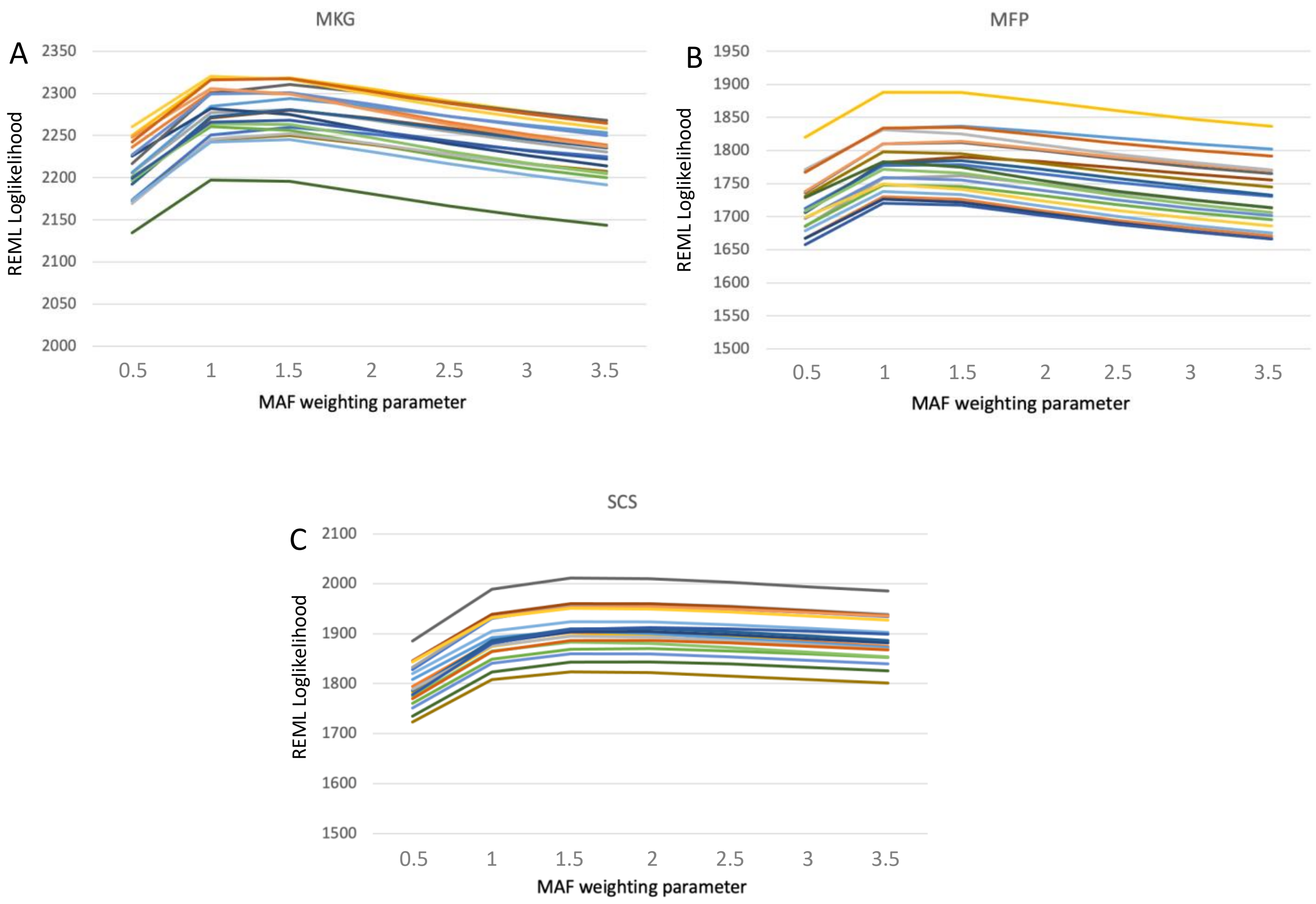

Figure S9: Relationship between GREML log-likelihood and the MAF weighting parameter using 80% of the dairy bull data

LD-pruned, significant SNPs were fitted as fixed effects for MKG and MFP. MAF weighting parameter is a (=b), where a and b are the same as defined in equation (2) and a=b=1 denotes non-weighted empirical GBLUP. Each line represents a replicate for a total of 20 replicates using 80% of individuals. MKG: milk yield; MFP: milk fat percentage; SCS: somatic cell score.

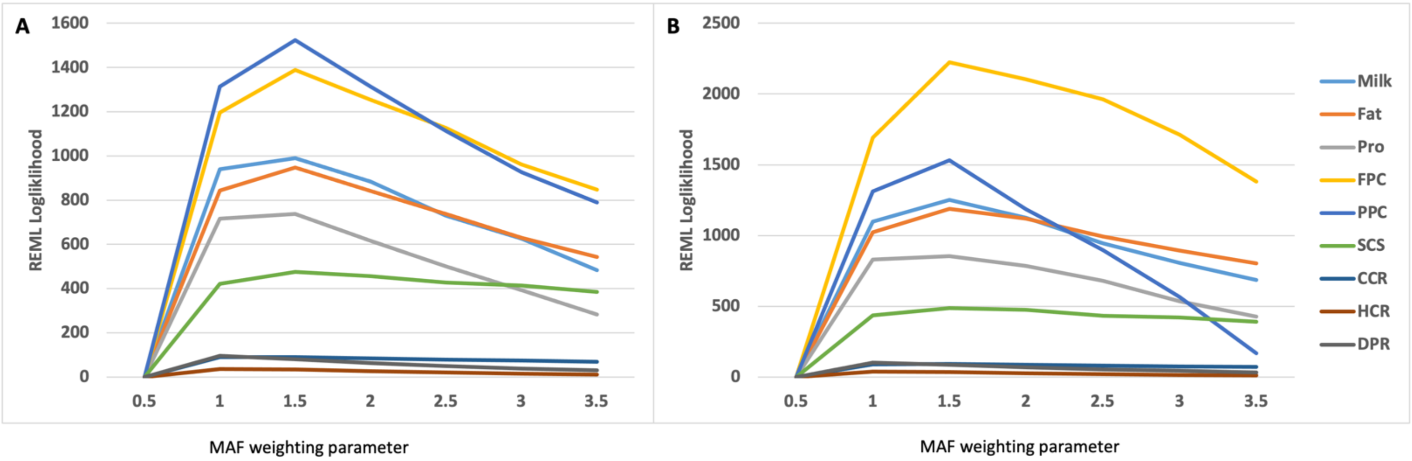

Figure S10: Relationship between GREML log-likelihood and the MAF weighting parameter using the CDCB dairy cow data

A: LD-pruned, significant SNPs were fitted as fixed effects for Milk, Fat, Pro, FPC, and PPC. B: No SNPs were fitted as fixed effect. REML log-likelihood values were shifted by a constant for each trait and cannot be compared between A and B. MAF weighting parameter is a (=b), where a and b are the same as defined in equation (2) and a=b=1 denotes non-weighted empirical GBLUP. FPC: fat percentage; PPC: protein percentage; Milk: milk yield; Fat: fat yield; Pro: protein yield; SCS: somatic cell count; CCR: cow conception rate; HCR: heifer conception rate; DPR: daughter pregnancy rate

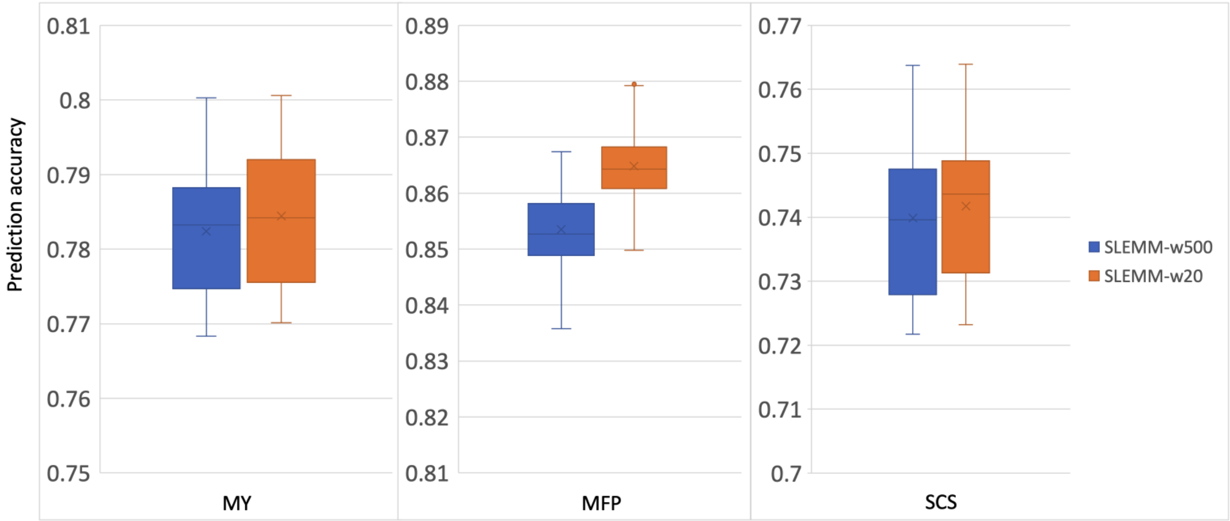

Figure S11. Predictive ability of SLEMM-WW-MAFopt with a window size of 20 or 500 SNPs for the dairy bull data

Data was randomly split into a training population (about 80% of individuals) and a validation population (about 20% of individuals) for 20 replicates. MY: milk yield; MFP: milk fat percentage; SCS: somatic cell score

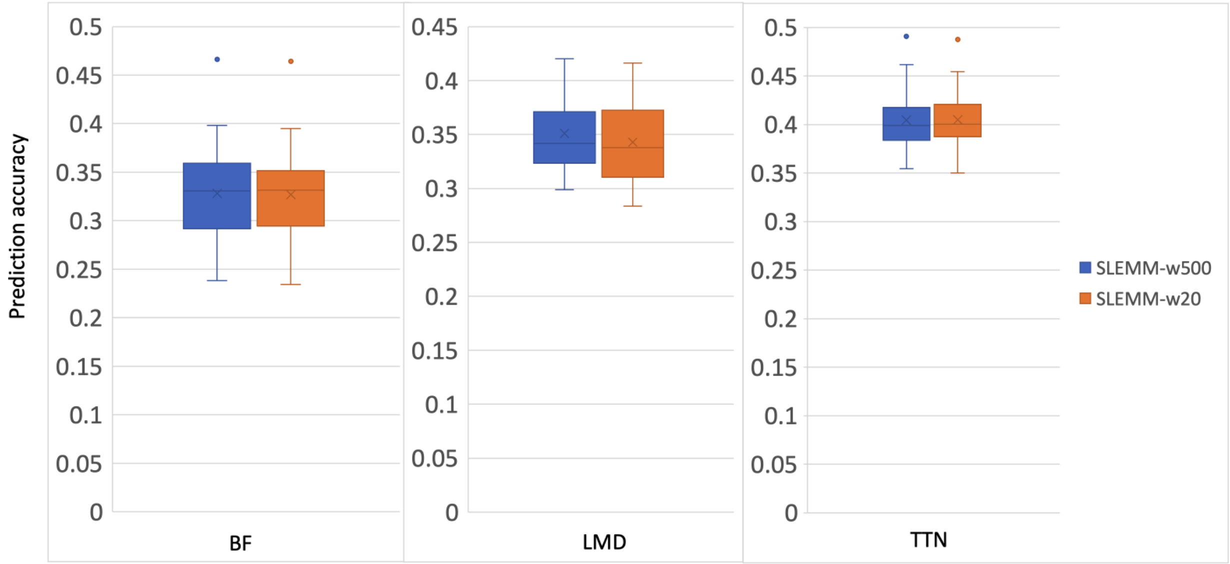

Figure S12. Predictive ability of SLEMM-WW-MAFopt with a window size of 20 or 500 SNPs for the Duroc pig data

Data was randomly split into a training population (about 80% of individuals) and a validation population (about 20% of individuals) for 20 replicates. BF: backfat; LMD: loin muscle depth; TTN: total teat number

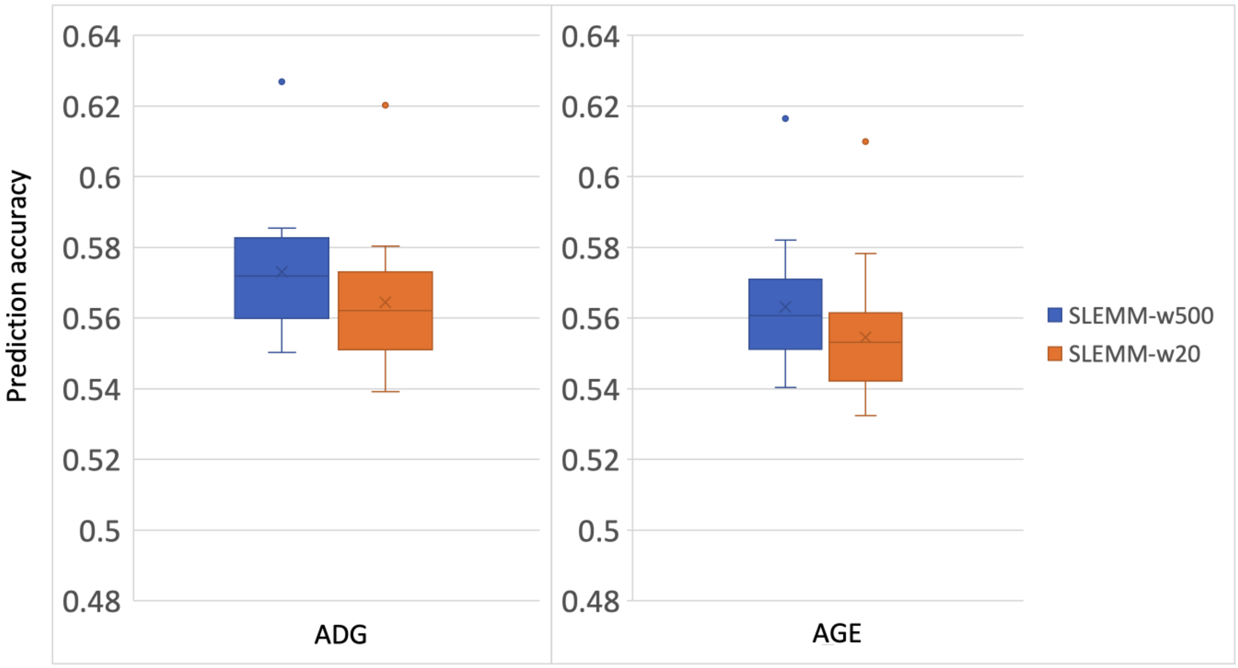

Figure S13. Predictive ability of SLEMM-WW-MAFopt with a window size of 20 or 500 SNPs for the four-breed pig data

Data was randomly split into a training population (about 80% of individuals) and a validation population (about 20% of individuals) for 20 replicates. ADG: average daily gain; AGE: off test age.

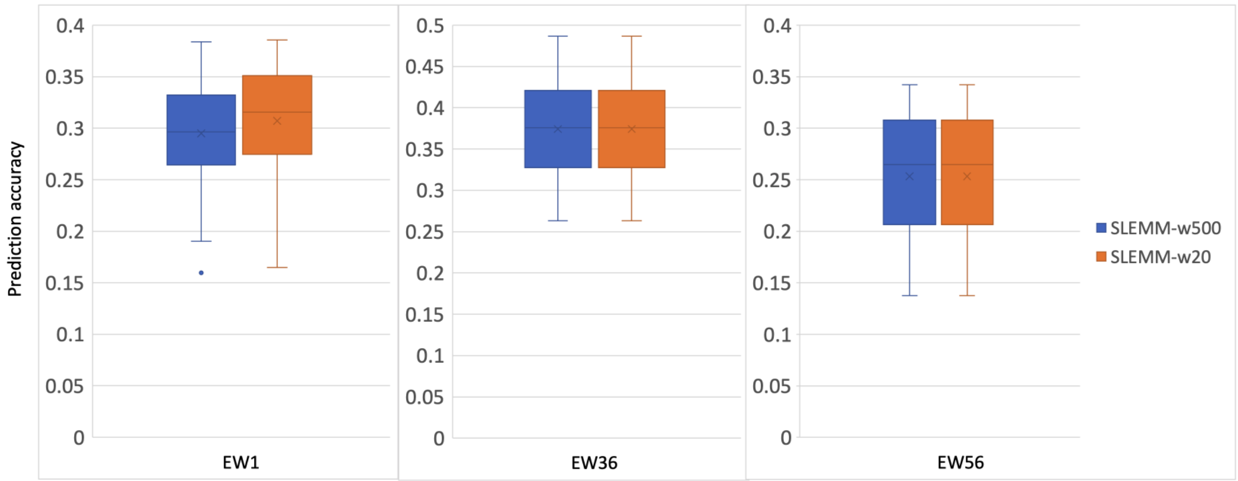

Figure S14. Predictive ability of SLEMM-WW-MAFopt with a window size of 20 or 500 SNPs for the chicken data

Data was randomly split into a training population (about 80% of individuals) and a validation population (about 20% of individuals) for 20 replicates. EW1: first egg weight; EW36: egg weight at 36 weeks old; EW56: egg weight at 56 weeks old

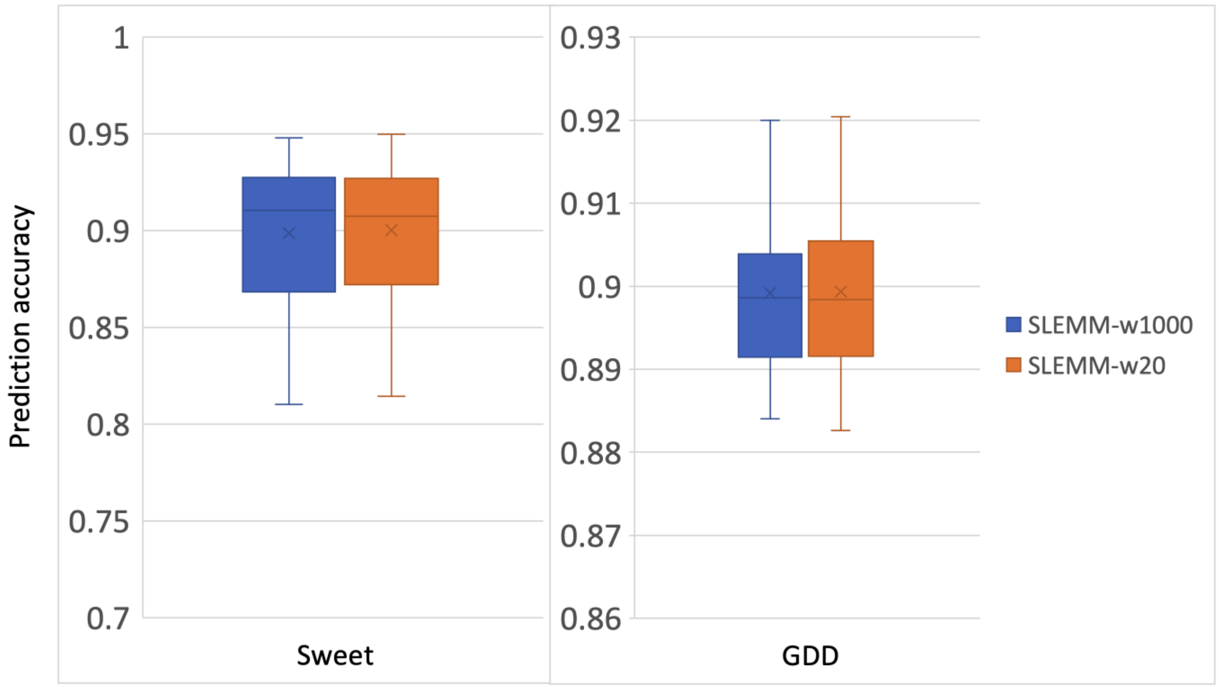

Figure S15. Predictive ability of SLEMM-WW-MAFopt with a window size of 20 or 1000 SNPs for the maize data

Data was randomly split into a training population (about 80% of individuals) and a validation population (about 20% of individuals) for 20 replicates. Sweet: kernel is sweet or starchy; GDD: growing degree days

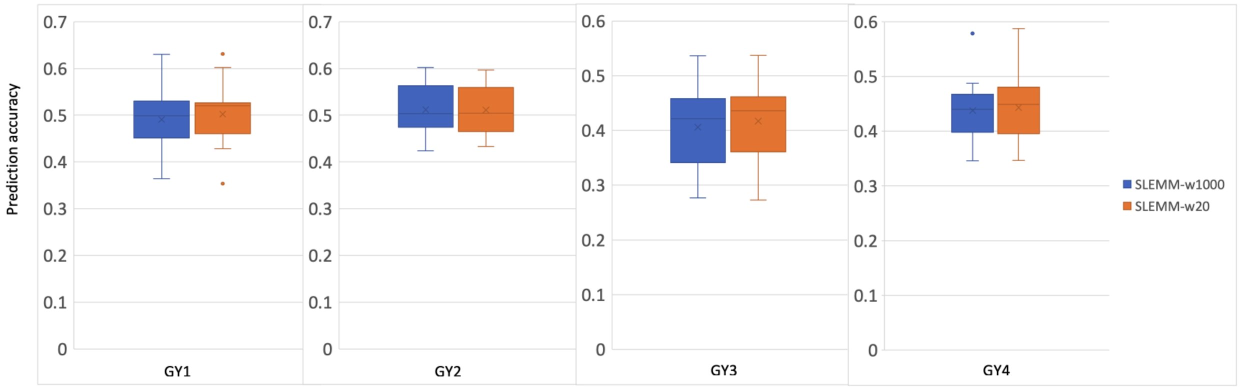

Figure S16. Predictive ability of SLEMM-WW-MAFopt with a window size of 20 or 1000 SNPs for the wheat data

Data was randomly split into a training population (about 80% of individuals) and a validation population (about 20% of individuals) for 20 replicates. GY1: grain yield in environment 1; GY2: grain yield in environment 2; GY3: grain yield in environment 3; GY4: grain yield in environment 4

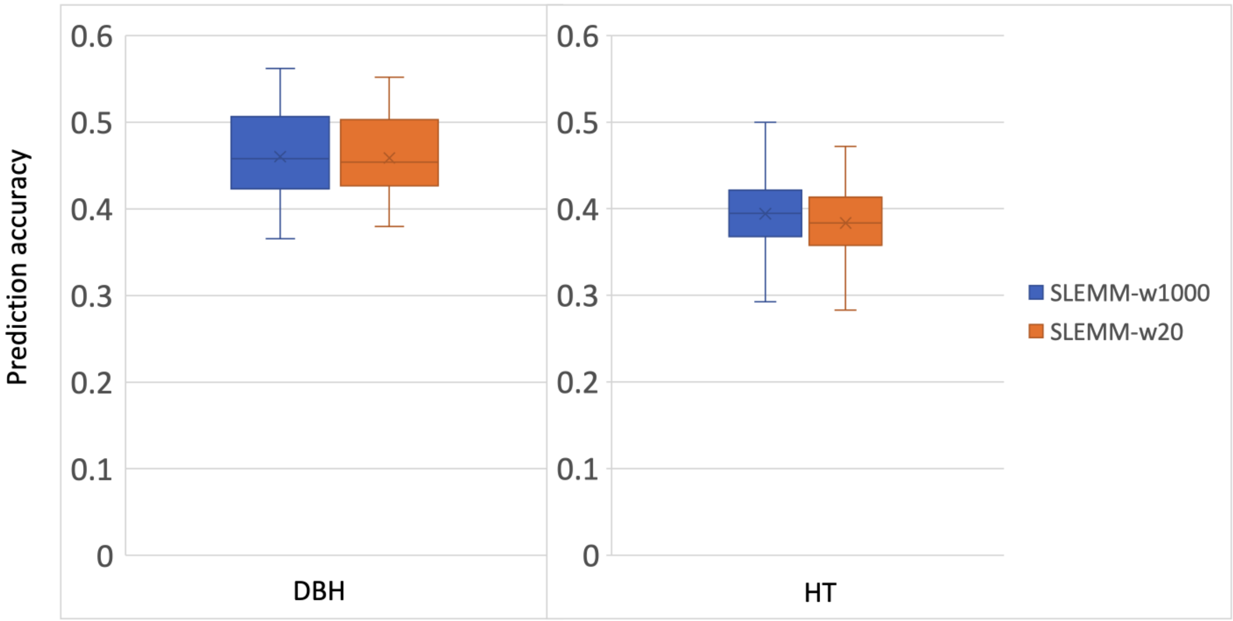

Figure S17. Predictive ability of SLEMM-WW-MAFopt with a window size of 20 or 1000 SNPs for the pine data

Data was randomly split into a training population (about 80% of individuals) and a validation population (about 20% of individuals) for 20 replicates. DBH: stem diameter; HT: total stem height

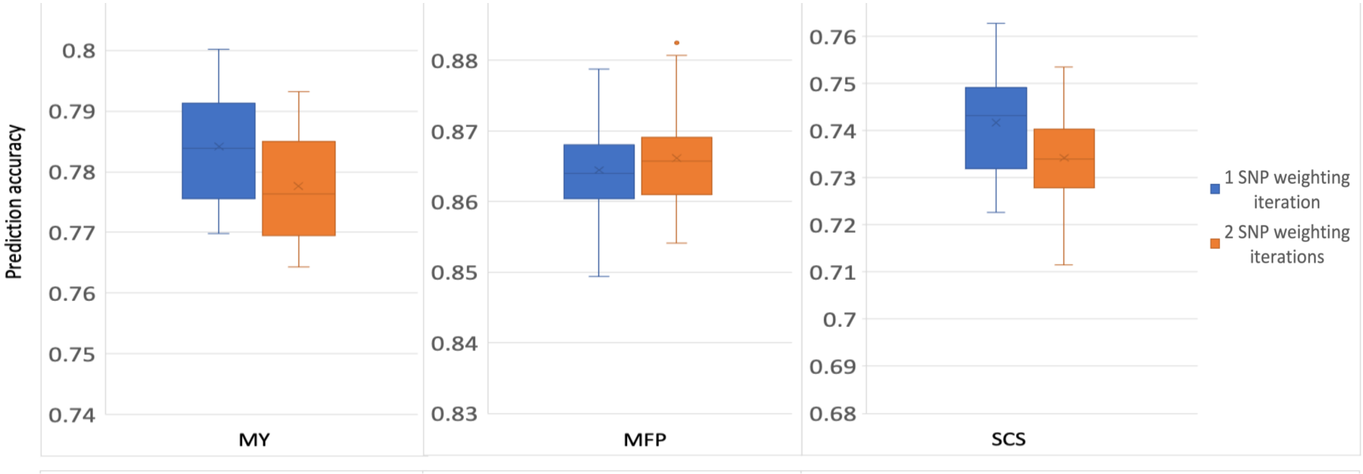

Figure S18. Predictive ability of window-weighted SLEMM (SLEMM-WW) using one or two SNP weighting rounds for the dairy bull data

Data was randomly split into a training population (about 80% of individuals) and a validation population (about 20% of individuals) for 20 replicates. MY: milk yield; MFP: milk fat percentage; SCS: somatic cell score
